# The haplolethality paradox of the *wupA* gene in *Drosophila*

**DOI:** 10.1371/journal.pgen.1009108

**Published:** 2021-03-19

**Authors:** Sergio Casas-Tintó, Alberto Ferrús

**Affiliations:** Instituto Cajal, Consejo Superior de Investigaciones Científicas, Madrid, Spain; Stowers Institute for Medical Research, UNITED STATES

## Abstract

Haplolethals (HL) are regions of diploid genomes that in one dose are fatal for the organism. Their biological meaning is obscure because heterozygous loss-of-function mutations result in dominant lethality (DL) and, consequently, should be under strong negative selection. We report an in depth study of the HL associated to the gene *wings up A* (*wupA*). It encodes 13 transcripts (A-M) that yield 11 protein isoforms (A-K) of Troponin I (TnI). They are functionally diverse in their control of muscle contraction, cell polarity and cell proliferation. Isoform K transfers to the nucleus where it increases transcription of the cell proliferation related genes *CDK2*, *CDK4*, *Rap* and *Rab5*. The nuclear translocation of isoform K is prevented by the co-expression of A or B isoforms, which illustrates isoform interactions. The corresponding DL mutations are, either DNA rearrangements clustered towards the gene 3’ end, thus affecting the genomic organization of all transcripts, or CRISPR-induced mutations in one of the two ATG sites which eliminate a subset of *wupA* products. The joint elimination of isoforms C, F, G and H, however, do not cause DL phenotypes. Genetically driven expression of single isoforms rescue neither DL nor any of the mutants known in the gene, suggesting that normal function requires properly regulated expression of specific combinations, rather than single, TnI isoforms. We conclude that the *wupA* associated HL results from the combined haploinsufficiency of a large set of TnI isoforms. The qualitative and quantitative normal expression of which, requires the chromosomal integrity of the *wupA* genomic region. Since all fly TnI isoforms are encoded in the same gene, its HL condition becomes unavoidable. These *wupA* features are comparable to those of *dpp*, the only other HL studied to some extent, and reveal a scenario of strict dosage dependence with implications for gene expression regulation and splitting.

## Author summary

Most species contain two copies of their genetic endowment, each received from each progenitor. If one of the duplicated genes is non-functional, the remaining copy may supply enough product as to cover the requirements for normal function or, alternatively, may reflect the insufficiency through a visible phenotype. In rare occasions, however, having one copy is so deleterious that causes lethality. These so called “haplolethal regions”, exist across species and represent an evolutionary paradox since they should have been subject to intense negative selection. The inherent difficulties to study haplolethals have precluded their study so far. Here, we analyzed the case of one of the five haplolethal regions of *Drosophila*, the one associated to the Troponin I encoding gene *wupA*, by measuring the transcriptional effects of mutations and chromosomal rearrangements affecting this gene. The data show that this haplolethality results from the combined insufficiency of a large number of Troponin I isoforms, which are functionally specialized, show interference and require the integrity of the native chromatin structure for their quantitatively regulated expression. These features unveil novel aspects of gene expression and, possibly, on evolutionary gene splitting. Also, haplolethals underscore the biological significance of protein dosage, in particular for functionally related products.

## Introduction

Diploid organisms are endowed with two genomic copies inherited from the parental generation. The gain or loss of small fragments from one or both of these genomic copies is referred to as segmental aneuploidies. When a single fragment is lost and the remaining copy is insufficient for normal biology, thus causing a phenotype, the segmental aneuploidy is referred to as haploinsufficient. In humans, haploinsufficient regions affecting to over 300 genes [1] are often associated to pathologies [e.g.: Parkinson’s disease [2]; bone marrow syndromes or myeloid malignancies [3] and autoimmune disorders [4], among others]. More rarely, haploinsufficiency may result in physiological advantages. In *S*. *cerevisiae*, a segmental haploidy increases stress resistance and ethanol production [5]. Interestingly, 3% of its 5900 genes are haploinsufficient when growing in enriched medium. The effect, however, can be alleviated by growing in minimal medium which suggests that the haploinsufficiency is due to low protein production [6]. Noticeably, 120 genes have never been recovered as deletions, suggesting that the absence of one copy of these genes may be lethal in the diploid phase of yeast [7]. Isolating such deletions is only feasible in diploid organisms by using suitable deficiencies and duplications. These regions are properly named as haplolethals (HL). Contrary to haploinsufficient regions, HL is a class of segmental aneuploidy whose nature remains largely unexplored due to the inherent difficulties to study them.

To justify a study on HL functions one should question how general HLs are? In the mouse, one HL function was identified by inducing segmental haploidy in ES cells followed by their injection in blastocysts. It was ascribed to the *t*-complex gene [8–10]. The HL function was assigned to a 3Mb region which includes several genes and, consequently, the correspondence with one or several genes was not unequivocally established. However, two other murine HL functions, also generated by treated ES cells, could be associated to the *Vegf* [11] and *Tcof1* [12] genes. Based on theoretical considerations, HL functions in the mouse are estimated as more likely to occur than haploinsufficient ones (see figure 6 in [1]). In humans, about 1% of all pregnancies include some form of deviation from normal diploidy [13], and chromosomal deletions of embryonic malformations data revealed that about 11% of the genome has never been recovered in the haploid condition or any other copy number variation [14–17]. Although small deletions in human genomes are recognized as largely underestimated [18], segmental aneuploidies have been detected in oocytes, 10.4%, raising to 24.3% by three days of embryogenesis, and declining to 15.6% in preimplantation blastocysts [19]. Thus, human genomes reveal their developmental instability, from meiosis to adulthood and, consequently, the need for continuous selection against aneuploidies. These observations could be taken as indirect evidence for deleterious HL functions in humans and its strong negative selection.

In *Drosophila*, the seminal work by the groups of Lindsley and Sandler in 1972 [20], based on segmental aneuploidies covering 85% of its genome, provided the first estimation of HL regions, up to 20, one of them being haplo-, as well as, triplo-lethal (Tpl). Since organism survival is inversely proportional to the extent of the genetic material deleted, the HL condition could result from the additive insufficiency of several adjacent genes. Thus, the estimated number of HL regions in flies has been reduced when smaller deletions have become available. A recent study of 793 small deletions covering 98.4% of the *Drosophila* genome indicates the number of HL regions as 5, including the Tpl [21]. The difficulty to study HL functions has precluded linking HL regions to specific genes, with only two exceptions. The *decapentaplegic* (*dpp*) gene includes an HL region which was functionally dissected from a recessive lethal phenotype using specific DNA fragments as transposons [22]. The HL region of *dpp* is referred to as Hin (haploinsufficient) in the corresponding literature. It spans 8 kb and it is thought to affect the five *dpp* transcripts. This Hin region seems refractory to insertions of the *P* type, but not to *hobo* type, transposons. Interestingly, the two 13 kb *hobo* inserts in the Hin region of *dpp* that had been reported, one of them landed in an intron, and the other did it in the 3’ untranslated region of exon 3 [23]. Thus, the *dpp* associated haplolethality is currently understood as a dosage insufficiency of the single encoded protein, the morphogen DPP a.k.a. BMP in vertebrates. Being the only HL region studied in *Drosophila* so far, we cannot evaluate how general the relationship between dosage insufficiency and haplolethality is. This issue is particularly relevant because *Drosophila* and most other genomes studied in some depth show a wide tolerance to dosage changes by means of transcriptional changes in functionally related genes outside the deleted region [24]. This effect is thought to reflect the robustness of genome networks [25]. This dosage tolerance renders even more intriguing the existence of haplolethals.

The *wings-up A* (*wupA*) gene is related to another HL region located at chromosome band 16F7 [26]. A haplolethal region implies the existence of dominant lethal loss-of-function mutations. Intriguingly, the mutational analysis of the region searching for the expected dominant lethal mutants (DL), had provided chromosomal rearrangements only, rather than point mutations, and all of them turn out to be located towards the 3’ end of the *wupA* transcription unit [27]. Remarkably, the Tpl region has proven also refractory to point mutations and only rearrangements were obtained [28]. Since the duplication required to isolate DL mutants in the 16F7 region, *Dp(1;3R)JC153*, also contains genes adjacent to *wupA*, the HL function could result from the combined insufficiency of several genes under the control of regulatory sequences located inside the *wupA* gene or, alternatively, from the haploinsufficiency of the *wupA* encoded protein, Troponin I (TnI). Although we have previously analyzed the biology of TnI beyond the muscle cells [27,29–33], we address here the functional specificity of its various isoforms with the aim of unraveling the genetic nature of the associated HL.

## Results

**Fig 1:**
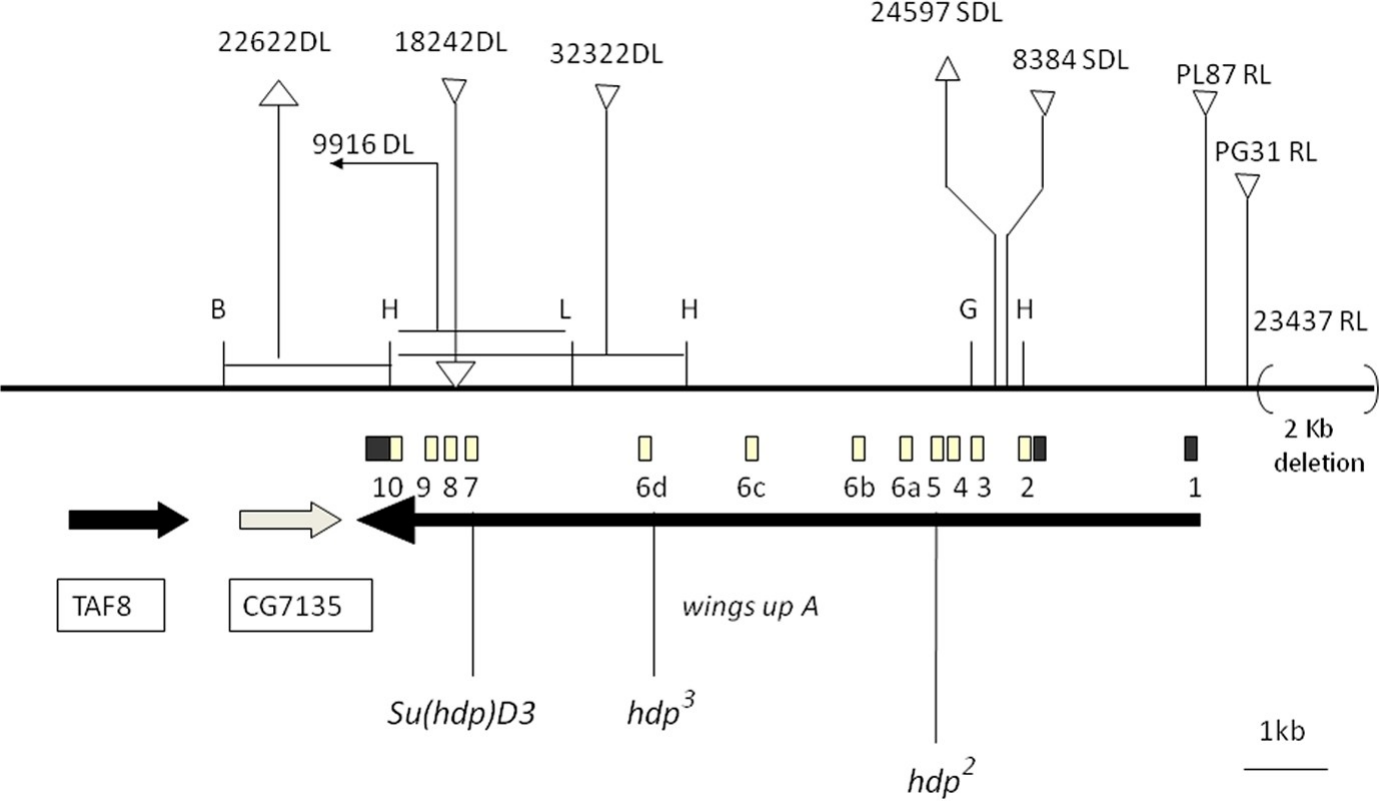
Physical map of the known mutations in the *wupA* gene. The diagram shows the relative positions of the *wupA* transcription unit (thick black arrow). Exons (1–10) are indicated by open (coding) and black (non-coding) boxes. Centromere is to the right. Transcription units *CG7135* and *TAF8* (a.k.a. *prodos*) locate distal to *wupA* and are indicate by thick arrows. Dominant lethal (DL) mutations are rearrangements whose breakpoint positions were determined by Southern blots with respect to enzyme restriction sites (B = *Bam*HI; H = *Hind*III; L = *Sal*I; G = *Bgl*II). *22622DL* is a deletion of the chromosomal segment 16F-18D. *32322DL* is an insertion of 23 Kb. *9916DL* is an inversion. The simplest of the DL mutations known to date, *18242DL*, is a 540 bp insertion in the intron between exons 7 and 8 (sequence deposited in EMBL X58188). Semidominant (SDL) rearrangements are also rearrangements. *24597SDL* is a deletion of about 0.4 Kb while *8384SDL* is an insertion of about 8 Kb. Recessive lethal (RL) are also rearrangements. *23437RL* is a 2 Kb deletion located at position -100 upstream of the transcription initiation site in *wupA*. *PG31RL* is a 11.279bp insertion located at -249 with respect to the same initiation site. *PL87RL* is another 10.691bp insert at position -30. All these RL rearrangements affect regulatory URE regions of *wupA* and severely reduce, but do not fully abolish, *wupA* transcription [31]. Point mutations are viable. Mutant *hdp*^*2*^ is an 116Ala>Val substitution in the constitutive exon 5; *hdp*^*3*^ is a one nucleotide change affecting the acceptor 3’ splice site for exon 6d which prevents the expression of TnI isoforms C, F, G and H (see **Fig 2**); and *Su(hdp*^*2*^*)D3* is an 188Leu>Phe substitution in the constitutive exon 7 which suppresses the *hdp*^*2*^ phenotype. Data are from Barbas *et al*.,1993; Prado *et al*., 1995; Prado *et al*., 1999.

**Fig 2:**
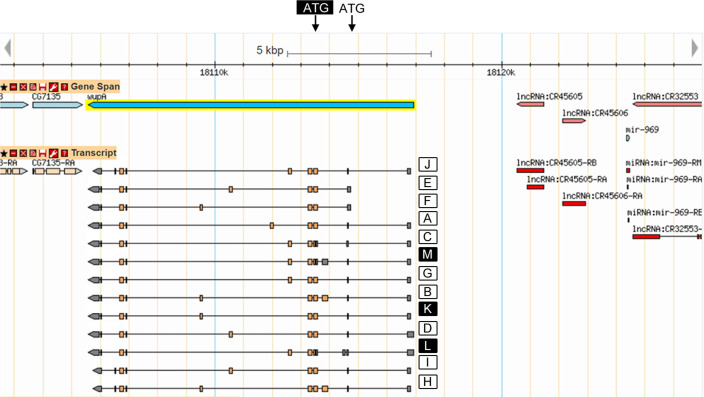
Transcriptional isoforms from *wupA* and adjacent genes. The diagram shows the 13 letter-coded transcripts emerging from the two ATG sites (white and black boxes). Note that the three black transcripts (K, L and M) share the same coding exons, thus, the gene yields 11 TnI protein isoforms. Data are available in FlyBase. Centromere is to the right. Note the proximal location of *lncCR45605*, *lncCR45606* and *mir-969* genes whose putative functional relationship with *wupA* is discussed in the main text.

This study deals mainly with the HL function located at the *Drosophila* chromosome band 16F7 where the gene *wupA* is located. Although the original data are available in previous reports [26,30,34], we summarize here the mutational analysis of *wupA* to facilitate reading this report and accommodate the novel data. Four categories of *wupA* mutations can be identified (**Fig 1**). Described from proximal to distal with respect to the centromere, the mutations are classified as Recessive lethals (RL), Semidominant lethals (SDL), Dominant lethals (DL) and Viables (V). The types RL, SDL and DL are discriminated by the viability of heterozygous females, 100% in RL, under 50% in SDL, and 0% in DL. While V mutations are single nucleotide changes, the RL, SDL and DL types are all chromosomal rearrangements. The transcriptional products from *wupA* result in two ATG translation sites (black and white boxes in **Fig 2**) yielding 13 different transcripts, A-M, which are shown letter-coded in **Fig 2**. Black transcripts K, L and M yield the same protein isoform, which reduces the total number of TnI isoforms to 11.

### The normal HL function at 16F requires the integrity of the *wupA* genomic region

**Fig 3:**
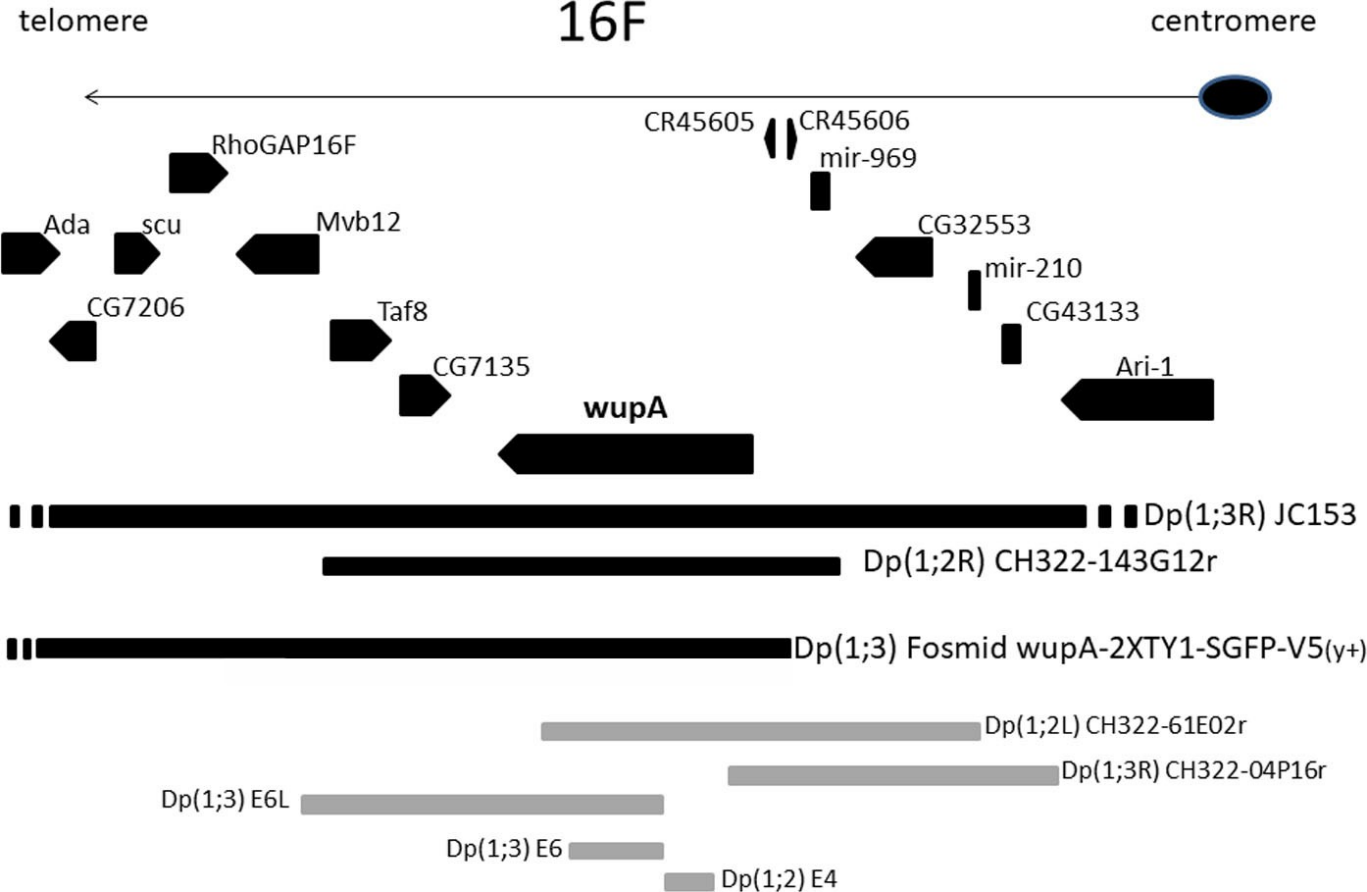
Functional map of duplicated chromosomal fragments of the *wupA* region. Known transcription units distal and proximal to *wupA* are shown as pointed boxes. Thick lines illustrate the functional extent of the various duplications tested. Those in black rescue all dominant lethal mutations while those in grey do not. Duplications *E6L*, *E6* and *E4* correspond to genomic fragments inserted in the corresponding chromosomes. *E6L* spans 8 Kb and rescue mutations in *Taf8* while E6 and E4 do not rescue any of the available mutants in the region. Duplications CH322 correspond to three pBAC constructs from which we obtained transgenic lines (see Materials and Methods). Only *CH322-143G12r* rescues DL mutants. This duplication is abbreviated as Dp1 in **Table 2**. The fosmid duplication *Dp(1;3) wupA-2XTY1-SGFP-V5* [35,75] rescues mutations in *Taf8* and, most but not all, mutations in *wupA* (see main text). The extent in the figure reflects the information available in the original report [35], although it does not rescue the lethality of *Ada3*^*7688*^ allele. This fosmid duplication is abbreviated as Dp2 in **Table 2**. Duplication JC153 spans 550 Kb and rescues all known mutants of the region. It originates from the insertional translocation *T(1;3)JC153*. This duplication is abbreviated as Dp3 in **Table 2**.

Over the years, a number of duplicated genomic fragments of the *wupA* region have become available (**Fig 3**). In order to establish a link between the HL function and genes in the region, each duplicated fragment was tested against all available mutants in *wupA* and adjacent genes, in particular all known DL mutants. Black fragments indicate rescue of DL and *wupA* mutants, and white ones indicate failure to do so. Duplication 3 [Dp3 = *Dp(1*,*3R) JC153*] is the largest fragment (>600 kb) and, as expected, it fully rescues all DL and the rest of *wupA* mutants. Duplication 1 [Dp1 = *Dp(1;2R) CH322-143G12r*], a pBAC with which we obtained a transgenic line, also rescues all *wupA* mutants. Duplication 2 [Dp2 = *Dp(1;3) wupA-2XTY1-sGFP-V5-preTEV-BLRP-3XFLAG*] is a fosmid construct [35]. Particularly relevant is the result that the combination of genomic fragments *E6L* plus *Dp (1;2L) CH322-61E02r* is still unable to rescue DL mutants even though it encompasses the entire *wupA* transcription unit, (albeit not in a continuous sequence) (**Fig 3**). If a regulatory sequence would be contained within the *wupA* transcription unit, it would not be able to operate in *trans*. Thus, the normal HL function at 16F7 requires the linear (*cis*) integrity of the *wupA* gene. In addition, the fact that ♀ DL/RL; Dp2/+ is lethal, but ♀DL/RL; Dp2/Dp2 is not, demonstrates that the RL and DL functions are functionally related and that they are dosage dependent on the products supplied by Dp2 (see foot notes in **Table 1**).

**Table 1:** Rescue by duplications.

Genotype	Rescue
♂ DL; Dp1/+	YES
♀ DL/+; Dp1/+	YES
♀ DL/DL; Dp1/+	NO
♀ DL/DL; Dp1/Dp1	<1%
♂ RL; Dp1/+	YES
♀ DL/RL; Dp1/+	YES
♂ hdp; Dp1/+	+ ^(a)^
♂ DL; Dp2/+	NO
♂ DL; Dp2/Dp2	YES
♀ DL/+; Dp2/+	YES
♀ DL/DL; Dp2/+	NO
♀ DL/DL; Dp2/Dp2	<1%
♂ RL; Dp2/+	YES
♀ RL/RL; Dp2/+	YES
♀ DL/RL; Dp2/+	NO
♀ DL/RL; Dp2/Dp2	YES
♂ hdp; Dp2/+	hdp ^(b)^

Notes.- Rescue determined from crosses in which a minimum of 100 progeny were counted (see Materials and Methods). Genotypes yielding escapers (<1% viability) consisted in short lived adults with normal gross morphology but unable to move properly or fully expand their wings and legs. **(a)** Dp1 rescues the wings up phenotype of *hdp*^*2*^ or *hdp*^*3*^ males. **(b)** Dp2 does not rescue the wing position phenotype in these alleles. See full genotypes and additional combinations in **S1 Table**.

Abbreviations: DL (dominant lethal) = *l(1)18242*^*DL*^ or *l(1)13193*^*DL*^; RL (recessive lethal) = *l(1)23437*; hdp = *hdp*^*2*^ or *hdp*^*3*^; Dp1 (duplication type 1) = *Dp(1;2R) CH322-143G12r*; Dp2 (duplication type 2) = fosmid, *wupA-2XTY1-sGFP-V5-preTEV-BLRP-3XFLAG*

The adjacent location of *mir-969* (http://flybase.org/cgi-bin/gbrowse2/dmel/?Search=1;name=FBgn0283471) to *wupA* and its inclusion in Dp1, a duplication that rescues all the DL mutants (**Fig 3** and **Table 1**), invited to explore its potential role in our HL function. Thus, *mir969* was expressed under the general driver *tub-Gal4*^*LL7*^ in normal, RL *l(1)23437* and V *hdp*^*3*^ backgrounds. In the first case, the overexpression of *mir969* throughout the body did not yield a visible phenotype. In the other two backgrounds, it failed to rescue or modify the phenotypes of either of these *wupA* mutations. Likewise, the depletion of *mir969* by means of expressing the construct *UAS-mCherry-mir969-sponge* in either of these three backgrounds, also failed to modify the *l(1)23437*^*RL*^ and *hdp*^*3*^ phenotypes nor to cause a DL condition. Most relevant, it should be noted that Dp2, which rescues the DL mutants, does not contain *mir-969* (**Fig 3**). Thus, we can conclude that *mir969* does not show evidences to suspect a functional interaction with *wupA* or its associated HL function. An equivalent reasoning allows excluding the other adjacent genes, *CG7135* and *Taf8*, since the genomic fragment E6L does not rescue any of the *wupA* mutants, while it does for *Taf8* ones (**Fig 3**).

Between *mir-969* and *wupA*, two additional genes have been identified recently which encode long-non-coding RNAs, *lnc45605* and *lnc45606* (**Fig 2**). Their *lnc* nature invites to consider their possible regulatory activity upon *wupA*. According to the physical data available, both of them are included within the three duplications, Dp1, Dp2 and Dp3, which rescue DL mutants (**Figs 2** and **3**). However, both *lnc* genes are also included in *Dp(1*,*2L)CH322-61E02r* which does not rescue any of the known *wupA* mutants. In addition, a deficiency that deletes both *lnc* genes, *Df(1)BSC352*, does not cause a DL condition (**S1 Fig**). Nevertheless, given the proposed regulatory activity of *lnc* genes in general [36], we tested this possibility by means of a gene complementation assay in females heterozygous for *Df(1)BSC352* over RL and V type *wupA* mutants (**S2 Fig**). *Df(1)BSC352* fails to complement the RL mutants, *l(1)23437*, *PL87* and *PG31*, and this lethality is rescued by Dp1, Dp2 or Dp3. The RL mutants are rearrangements (**Fig 1**) that alter the structure of the URE regulatory region of *wupA* [31], and this could justify the lack of complementation by *Df(1)BSC352*. However, that deficiency complements the V type mutants *hdp*^*2*^ and *hdp*^*3*^, while the RL mutants do not. The differential complementation of *wupA* mutants could indicate that *Df(1)BSC352* deletes selected regulatory elements of *wupA* expression, including *lnc45605* and *lnc45606*, while leaving others intact. Actually, enhancers corresponding to the IRE region [31] are not deleted in *Df(1)BSC352*. In the absence of genetic tools that could allow a more thorough functional analysis of these *lnc* genes, we must consider their putative role on *wupA* regulation as still open. If future experiments would proof that role, it is clear already that *lnc* haploinsufficiency is independent from haplolethality because the heterozygous *Df(1)BSC352/+* is not DL (see **Discussion**).

In addition to the HL functions linked to *wupA* and *dpp* genes, there are other genomic regions for which the deletion analysis indicates HL conditions. We explored the possible interaction between the *wupA* HL and the rest of the putative HL functions, excluding the Tpl region due to the lack of suitable genetic tools (**S1 Table**). We tested genotypes carrying a DL mutant at *wupA* and duplications that rescue the other reported HL regions [37,38]. Likewise, we tested deficiencies that uncover these other HL regions with duplications that cover *wupA*. None of these combinations modified the corresponding HL phenotypes. Thus, we conclude that there is no evidence, at this point, to suggest a functional link among HL functions within the *Drosophila* genome. They do share, however, the coding of multiple transcripts and, in the known cases of *dpp* and *wupA*, the corresponding DL mutants are rearrangements broken in non-coding sequences which affect most, if not all, gene transcripts. Is the biology of HL genes substantially different from that of non-HL counterparts?

### Single transcripts from *wupA* rescue neither DL nor *wupA* mutants

UAS constructs were generated for each *wupA* transcript and tested with the general driver *tub-Gal4*^*LL7*^ in order to assay if the overexpression of any of them would rescue mutants located in this gene. The data show (**Table 2**) that none of the white transcripts, nor the single black one tested, rescue any of the *wupA* mutant types including the DL type. The rescue of *hdp*^*3*^ by isoforms B and E is partial since they restore wing position, albeit not flight, in about 25% of adults only. At least two different transgenic insert lines were tested per UAS construct.

**Table 2:** RESCUE OF *wupA* MUTANTS.

	DL		SDL	RL			V	
	*18242*^*DL*^	*13193*^*DL*^	*24597*^*SDL*^	*23437*^*RL*^	*PL87*^*RL*^	*PG31*^*RL*^	*hdp*^*2*^	*hdp*^*3*^
A	-	-	-	-	-	-	-	-
B	-	-	-	-	-	-	-	+^(b)^
C	-	-	-	-	-	-	-	-
D	-	-	-	-	-	-	-	-
E	-	-	-	-	-	-	-	+^(b)^
F	-	-	-	-	-	-	-	-
G	-	-	-	-	-	-	-	-
H	-	-	-	-	-	-	-	-
I	-	-	-	-	-	-	-	-
J	-	-	-	-	-	-	-	-
K	-	-	-	-	-	-	-	-
**Dp1**	+	+	+	+	+	+	+	+
**Dp2**	+ ^(a)^	+ ^(a)^	+	+	+	+	-	-
**Dp3**	+	+	+	+	+	+	+	+

Notes.—The *wupA* transcripts are depicted in white or black (A-K) according to their corresponding ATG site (see **Fig 2**). Every isoform was driven as UAS constructs by *tub-Gal4* and a minimum of 100 offspring adults per cross were counted. Black transcript isoforms K, L and M encode the same protein, thus only K was assayed. (a) Rescue is dose dependent; one copy of Dp2 can rescue ♀DL/+ but two copies are needed to rescue ♂DL or ♀DL/DL. In the last case, only escapers are recovered as adults (<1% viability). The same driver was used to co-overexpress several transcripts (C+H, J+F and J+G) but none of these combinations rescued *18242*^*DL*^ neither in males nor in heterozygous females. (b) Rescue of *hdp*^*3*^ by either of these two transcripts was obtained for 25% (B) and 20% (E), respectively. The rescue, however, affected to wing position but not to flight. The joint overexpression of C and H also failed to rescue *hdp*^*3*^.

Abbreviations: Dp1 (duplication 1) = *Dp(1;2R) CH322-143G12r*; Dp2 (duplication 2) = fosmid, *wupA-2XTY1-sGFP-V5-preTEV-BLRP-3XFLAG*; Dp3 (duplication 3) = *Dp(1;3R) JC153*

We also tested three relevant genomic duplications with the same set of *wupA* mutants in order to compare with the results obtained with the UAS constructs (**Table 2**). Duplication 2 yielded interesting rescue effects. It shows dose dependence in the rescue of DL mutants. One copy of Dp2 is sufficient to rescue DL/+ heterozygous females, while two copies are needed to barely rescue DL/DL homozygous adult females. Two copies are also needed to rescue DL males (see footnotes in **Table 2**). Another interesting feature of Dp2 is its failure to rescue the V type mutants *hdp*^*3*^ and *hdp*^*2*^. This last result demonstrates that Dp2, although able to rescue DL mutants, does not supply the full set of normal functions for *wupA*. To a lesser extent, Dp1 also show insufficient supply of normal *wupA* function since two copies can yield only 1% of adult DL/DL female escapers while one copy is sufficient to rescue wing position in *hdp* alleles (**Table 1**).

**Fig 4:**
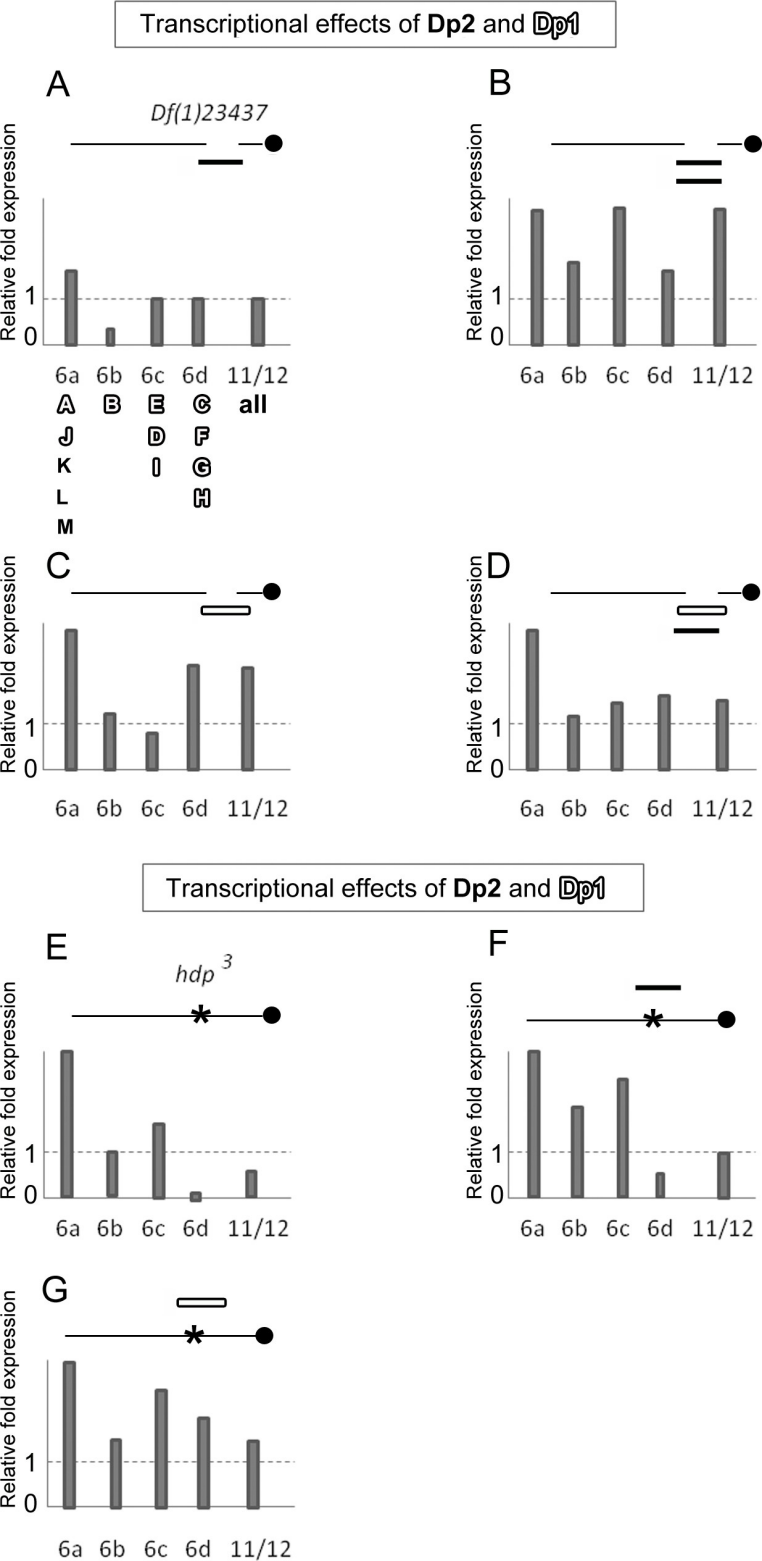
Transcriptional effects of duplications Dp1 and Dp2 covering *wupA* region. A-D) Effects on *l(1)23437* background. We tested the transcriptional efficiency of these two duplications using exon 6 specific primers in qRT-PCR assays on severe hypomorph (*23437*) males. The *wupA* transcripts identified by each exon-specific primer are indicated in **A**. Dotted line indicates the normalized levels of controls. Note that one dose of *fosmid* (Dp2) fails to produce normal levels of isoform B and two doses do not duplicate linearly the transcription of all isoform. A similar transcriptional heterogeneity is observed with *Dp(1;2R)CH322-143G12r* (Dp1) in one dose or in combination with Dp2. **E-G) Effects on *hdp***^***3***^ **background**. The same heterogeneity is detected on this background. Note, however, that, in general, the transcriptional efficiency of Dp1 is higher than that of Dp2. This is consistent with the rescue data shown in **Table 2**.

Since two of the duplications, Dp1 and Dp2, showed differential rescue effects with respect to Dp3, this feature invited to analyze the transcriptional properties of these inefficient duplications. To that end, we used qRT-PCR to measure the transcriptional yield of adult males with Dp1 and Dp2 in *l(1)23437* and *hdp*^*3*^ backgrounds. These backgrounds and the designed exon specific probes facilitated the identification of *wupA* transcripts to the extent possible (**Fig 4**). In the *l(1)23437* background (**Fig 4A and 4D**), the heterogeneity in the relative levels of the various transcripts, either in the presence of one or two copies of Dp2 or in combination with Dp1, becomes evident. In the *hdp*^*3*^ background (**Fig 4E–4G**), the phenomenon of quantitative transcriptional heterogeneity is also detected. Even the point mutation *hdp*^*3*^ that, altering the splice site for exon 6d should have affected the C, F, G and H red transcripts only, yields overexpression of white A, J, and black K, L and M products (**Fig 4E**). Neither Dp1 nor Dp2 can normalize this *hdp*^*3*^ caused overexpression. These transcriptional data uncover a fine quantitative regulation of the *wupA* transcriptional expression that was unsuspected hitherto. Not only the genomic duplications do not provide the expected levels of transcripts, but also a splice site mutation as *hdp*^*3*^ seems to alter the expression of transcripts that do not include the affected exon, 6d. In addition, these data also suggest that the *wupA* encoded products, isoforms of Troponin I (TnI), may not be functionally equivalent.

### The *wupA* products interact and are functionally diverse

In a previous study we had shown that the attenuation of *wupA* expression by means of RNAi or its overexpression using the TnI overexpressing construct *PBac(WH)f06492* yield noticeable effects on cell proliferation [33]. This overexpression is synergistic with standard oncogenes such as *Ras*^*v12*^, *Notch* or *lgl*, while its attenuation largely suppresses their tumor overgrowths. Following the generation of UAS constructs for single *wupA* transcripts, we analyzed the effects of their individual overexpression in order to determine if all TnI isoforms play similar functions in cell proliferation or if, by contrast, they show functional specificities.

**Fig 5:**
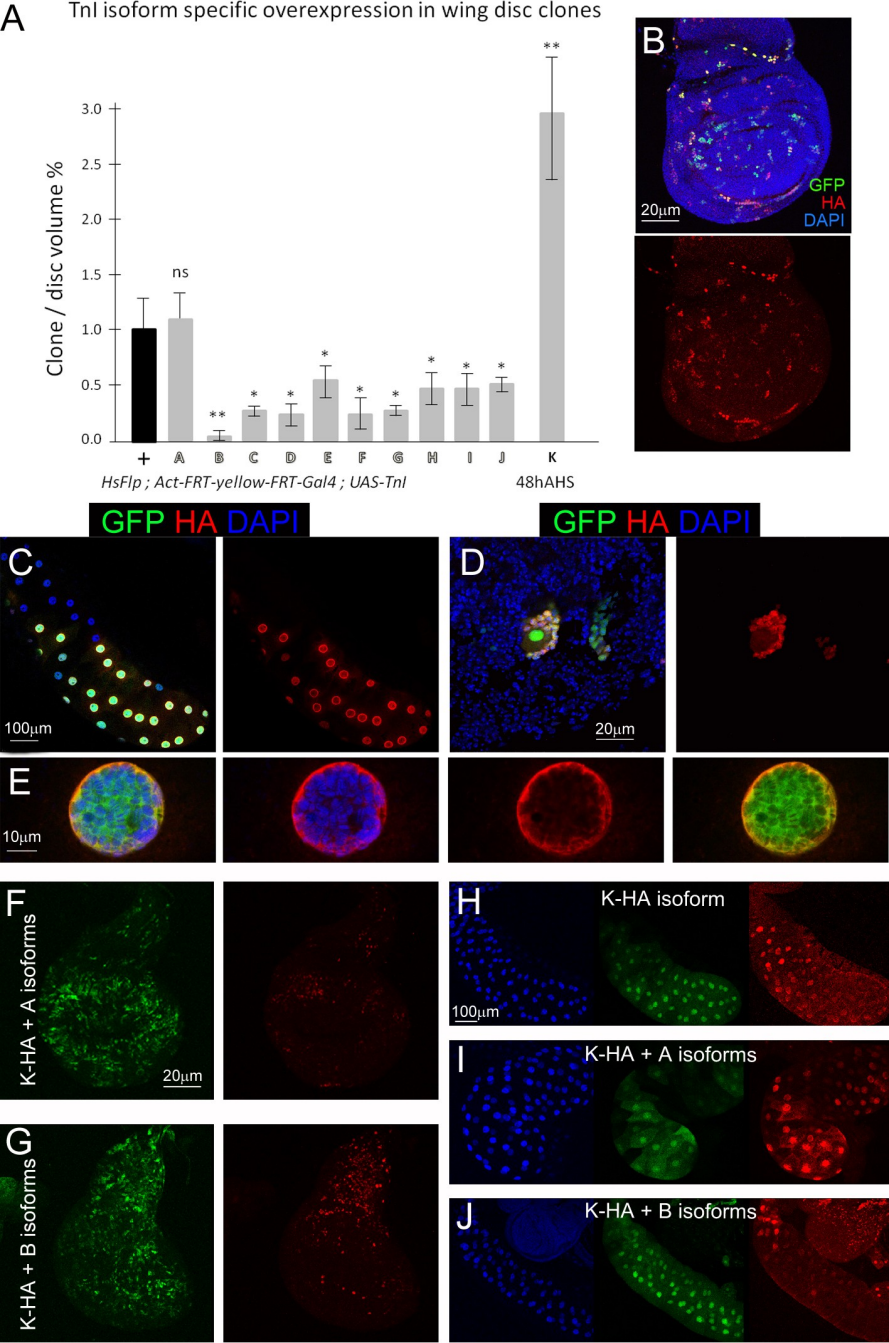
TnI isoforms are functionally different and interacting. A) Cell proliferation effects of single TnI isoforms overexpression. Somatic clones were induced in the larval wing discs (genotype: ♀♂ *hsFlp; act-FRT-yellow-FRT-Gal4/+; UAS-TnI*^*isoformX*^*/+*) screened 48h after heat shock (48AHS). Note that, with the exception of A, all white isoforms reduce cell proliferation. By contrast, the black isoform tested (K), yields a significant proliferation increase. Since all black isoforms (K, L and M) yield the same protein, only one was tested. **B-E) Nuclear localization of isoform K. B)** Images of larval wing disc somatic clones expressing the HA-tagged K isoform (red) and the cell reporter GFP to identify the clones. Nuclei are stained by DAPI (blue). **C)** Equivalent clones in the salivary glands and **D**) the neuroblast descendants in the central nervous system. **E**) Lower row of panels show one salivary gland nucleus stained for DAPI (blue) and TnI (red). Note the nuclear localization of K-HA, mostly in the periphery. **F-J) The nuclear localization of isoform K is interfered by isoforms A or B**. Equivalent clones co-expressing TnI isoforms K-HA and A or B in wing imaginal discs (**F, G**) or salivary glands (**H-J**). The nuclear localization of isoform K is efficiently prevented by isoform A and, to a higher extent, by isoform B.

To that end, we generated somatic clones in larval wing discs and quantitated clone size with respect to wing disks of sibling controls expressing an innocuous UAS construct (**Fig 5A**). The data reveal at least three different phenotypic effects on cell proliferation: 1) No effect (transcript A), 2) Underproliferation to various degrees (transcripts B-J), and 3) Overproliferation (transcript K). Thus, different TnI isoforms affect cell proliferation and/or survival in different ways. While most isoforms reduce cell proliferation, isoform K causes a strong proliferative effect when overexpressed. Since the three black transcripts K, L and M encode the same protein, testing only one of them is justified.

Also in a previous study, we showed by immunodetection that TnI traffics to the cell nucleus as a function of cell cycle status [32]. Since isoform K causes overproliferation and we had shown previously that the generalized overexpression of *wupA* triggers overexpression of cell division related genes [33], we investigated if isoform K, by itself, could have the capacity to translocate to the nucleus and trigger transcriptional changes in cell proliferation related genes. To that end, we created an HA-tagged version of isoform K and expressed it in wing disc somatic clones, salivary glands and neuroblasts. The HA tag is detected in the nucleus in all three cell types (**Fig 5B–5E**). Benefiting from the large size of salivary gland nuclei, the HA-K signal can be clearly identified in the periphery of the nucleus (**Fig 5E**). This localization of isoform K is consistent with the immune detection that we reported using a polyclonal TnI antibody (see figure 1 in [39]).

In addition, we tested the eventual changes in the expression of a set of genes involved in the control of cell proliferation by qRT-PCR (**S3 Fig**). We used RNA from larvae overexpressing isoform K (genotype: *tub-Gal4*^*LL7*^ *> UAS-K*). Consistent with our previous study, a subset of these genes are overexpressed when the isoform K is in excess. In particular, *CDK2*, *CDK4*, *Rap* and *Rab5* exhibited significant overexpression with respect to controls (genotype: *tub-Gal4*^*LL7*^ *> UAS-LacZ*). *CD2* and *CDK4* are well known inducers of cell cycle entry [40]. For example, *CDK2* mediates *Myc* induced cell proliferation through its association with Cyclin E [41,42], while *CDK4* plays equivalent roles through its association with Cyclin D [43,44]. Likewise, *Rap*, a Fizzy-related protein, regulates cell proliferation [45], and *Rab5* contributes to proliferative cell signaling through the titration of EGFR [46], among other functions.

Given the strong effect of isoform K on cell proliferation (**Fig 5A**) and its nuclear localization, we questioned if its generalized overexpression could yield a visible phenotype. Two constructs were tested, HA-K and non-tagged K, driven by *tub-Gal4*^*LL7*^. In both cases, the genotype was adult lethal. Other drivers with more restricted domains of expression, *en-Gal4* and *rn-Gal4*, yielded poorly viable adults (<10%) with various morphological abnormalities in wings and legs (**S4 Fig**). Thus, we conclude that isoform K overexpression is deleterious.

In view of the diversity of TnI effects on cell proliferation (**Fig 5A**), we questioned if the function of a given transcript could be modified by others from the same gene. As an example, we chose the nuclear localization of the K isoform (black) under the co-expression of white isoforms. Thus, we generated somatic clones expressing HA-K along with A or B isoforms. The data show that both white isoforms prevent the nuclear localization of K in imaginal discs and salivary glands, being the A effect weaker than that of B (**Fig 5F–5J**). To further investigate possible additional examples of isoform interference, we tested the adult lethality caused by the excess of K (genotype: *tub-Gal4*^*LL7*^ > K), but generated from females heterozygous for the RL mutant *l(1)23437*. In this case, the overexpression of K is no longer lethal and females *l(1)23437/+; tub-Gal4*^*LL7*^*/K* are 100% viable. This result suggests that the relative depletion of TnI isoforms caused by the *23437* mutant in the maternal oogenesis or early embryogenesis could alleviate the deleterious overexpression of isoform K. The suppression of the lethality due to K excess is also observed when the maternal progenitor is heterozygous for other RL mutants, *PL87* and *PG31*. The V mutants *hdp*^*3*^ and *hdp*^*2*^ also suppress the K-dependent lethality, always when the RL or V mutants come from maternal, not paternal, origin. As shown above (**Fig 4E**), *hdp*^*3*^ eliminates isoforms C, F, G and H, and *hdp*^*2*^ is a point mutation in a constitutive exon (**Fig 1**). On the other hand, the co-overexpression of K with isoforms B or A maintains the lethality. Finally, as shown in **Table 2**, isoforms B or E can rescue, to some extent, the wings up phenotype of *hdp*^*3*^. That is, the depletion of C, F, G and H isoforms can be compensated, at least in part, by the excess of B or E (see foot note in **Table 2**).

**Fig 6:**
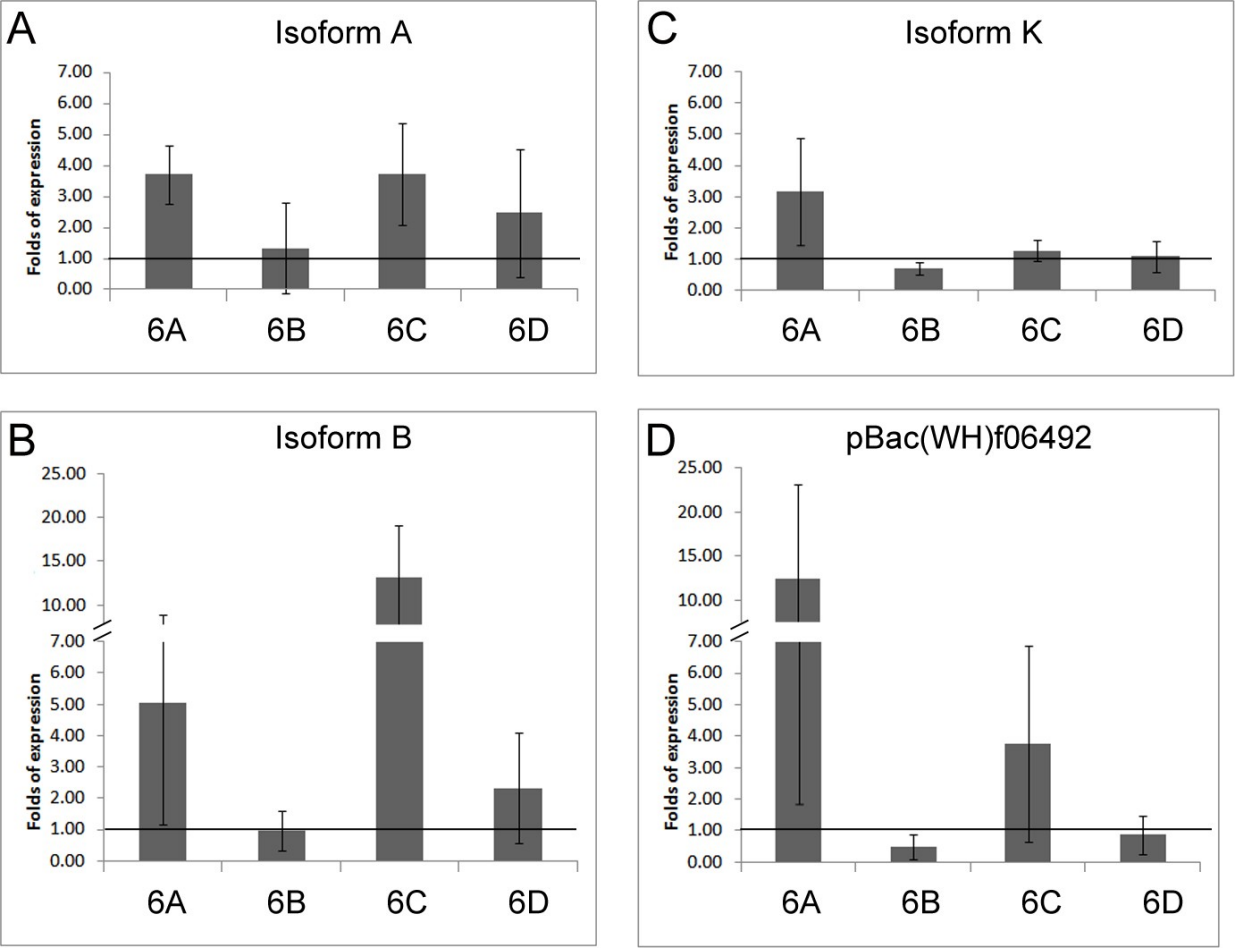
Transcriptional effect of driving single TnI isoforms. **A-C)** Overexpression of isoforms A, B and K. **D)** Overexpression of the Exelxis construct for *wupA*. Note the systematic lack of effect upon the expression of isoform B which could suggest a regulatory role for this isoform (see main text). Genotype: ♂ *tub-Gal4*^*LL7*^>*UAS-TnI*^*isoformX*^. Primers from exons 6a-d identify TnI isoforms as indicated in **Fig 4A**. Transcriptional levels are normalized to sibling controls ♂ *TM3/UAS-TnI*^*isoformX*^.

Beyond these in vivo phenotypes, we investigated the transcriptional effects of overexpressing specific TnI isoforms. The rationale being that, if there is phenotypic interference among isoforms, there could be transcriptional interference also (**Fig 6**). The data show that the overexpression of isoform A (**Fig 6A**), in addition to yield its own excess, as revealed by probe 6a, it also causes excess of probe 6c detected isoforms, E, D and I. Surprisingly, driving isoform B (**Fig 6B**), yields a significant excess of most other isoforms except of itself. This paradoxical result could indicate, perhaps, a regulatory effect of isoform B on *wupA* transcription but the issue was not investigated further. The equivalent experiment driving isoform K (**Fig 6C**) seems to be rather specific for that isoform as revealed by probe 6a. The transcriptional effect was also reproduced by driving the HA-tagged version of isoform K (**S5 Fig**). Finally, we included in this set of experiments the transcriptional effects of the *PBac(WH)f06492* since this UAS-dependent construct had been shown to cause strong proliferation effects synergic with standard oncogenes [32], as mentioned above. The data show (**Fig 6D**) that this construct elicits the strong overexpression of 6a revealed isoforms, which include isoform K, and, to a lesser extent, the 6c detected isoforms. This transcriptional effect is consistent with the cell proliferative phenotype observed with isoform K overexpression (**Fig 5A**).

Taken together, these experiments uncover a wide range of functional interactions among TnI isoforms operating throughout development and cell types. Presumably, the repertoire of interactions will extend beyond the cases experimentally analyzed here. Their large combinatorial number, however, precludes an exhaustive analysis at this time. It seems that the normal biology of the *wupA* gene consists of a collection of diverse, albeit interacting, functions achieved by the ensemble of encoded TnI isoforms. Likely, the expression of these isoforms will be subject to a tight quantitative regulation. Could a combined depletion of all or several TnI isoforms account for the HL function at 16F?

### Targeting a subset of *wupA* products causes a DL phenotype

**Fig 7:**
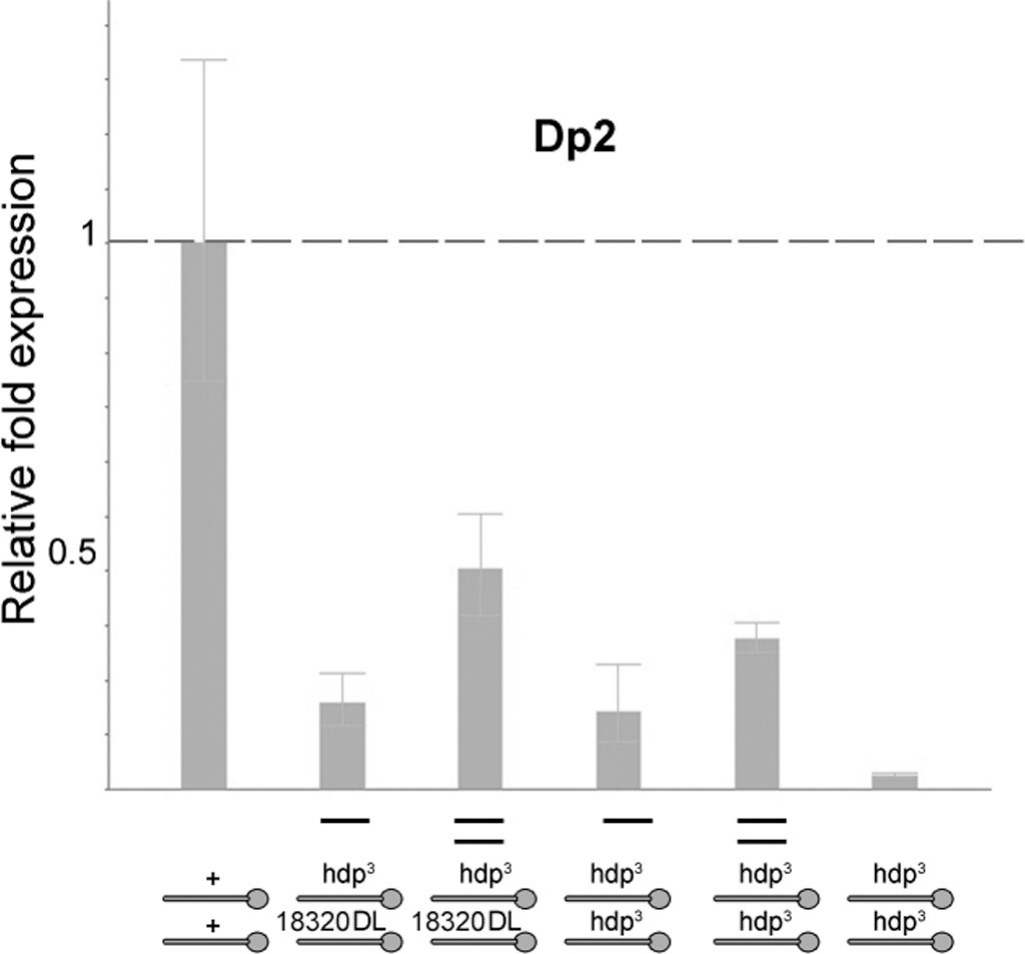
Transcriptional effects of the dominant lethal mutant allele *18320C*. Genotypes are indicated below each histogram. Primers from exon 6d were used to monitor isoforms C, F, G and H. Transcriptional levels are normalized to sibling controls. Note that *Dp2*, either in one or two doses, although it rescues the dominant lethality of *18320C*, denoted as DL for brevity, it does not supply the normal levels of 6d containing isoforms, 25% roughly. Also, since ♀ *hdp*^*3*^*/hdp*^*3*^*; Dp2/+* and ♀ *hdp*^*3*^*/18320C; Dp2/+* yield the same levels of these transcripts, it follows that *18320C* is null for these isoforms. By extension, since *18320C* is a point mutation in the white ATG site, this mutant should be also null for the rest of white TnI isoforms.

The functional diversity and interactions of TnI isoforms invited to consider their haploinsufficiency as the cause of the HL phenomenon. To address this possibility, we obtained a point mutation (A>C) at the white ATG site by means of the CRISPR/Cas9 system (see Materials and Methods and **S6 Fig**). Two independent mutations, *18230C* and *18230B*, were isolated and tested for their viability effects in combinations with DL, RL and V mutants and the same three genomic duplications used in previous experiments. The key data for allele *18320C*, which was validated by sequencing, are shown in **Table 3** and **Fig 7**.

**Table 3:** FUNCTIONAL PROPERTIES OF A Dominant Lethal MUTATION IN THE white ATG SITE OF *wupA*.

Genotype	Rescue/Phenotype
*♂ 18320C; Dp3/+*	+
*♂ 18320C; Dp2/+*	+
*♂ 18320C; Dp1/+*	+
*♀ 18320C/+; +/+*	Lethal
*♀ 18320C/18320C; Dp1/Dp1*	+
*♀ 18320C/18320C; Dp1/+*	Lethal
*♀ 18320C/18242*^*DL*^ *; Dp3/+*	<10%
*♀ 18320C/18242*^*DL*^ *; Dp2/+*	Lethal
*♀ 18320C/18242*^*DL*^ *; Dp1/+*	Lethal
*♀ 18320C/23437*^*RL*^ *; Dp3/+*	+
*♀ 18320C/hdp*^*3*^ *; Dp2/Dp2*	hdp

Notes.- Assays were carried out with two independent CRISPR/Cas9 mutations (ATG> XYZ) induced on the white ATG site (see **Fig 2**). Table shows the results for mutant *18320C* which were identical to those from *18320B*. Mutant *18320C* was validated by PCR and sequencing (see Materials and Methods and **S5 Fig**). A minimum of 100 adult offspring per cross were counted. The data indicate that *18320C* and *18320B* belong to the DL type of *wupA* mutants.

Abbreviations: Dp1 (duplication type 1) = *Dp(1;2R) CH322-143G12r*; Dp2 (duplication type 2) = fosmid, *wupA-2XTY1-sGFP-V5-preTEV-BLRP-3XFLAG*; Dp3 = *Dp(1;3R)JC153*

Females heterozygous for *18230C* show 0% viability, thus proving its DL condition. This lethality is rescued by the three genomic duplications, Dp1, 2 and 3, when tested in males. In homozygous females, however, Dp1 shows the same dosage dependence that Dp2 had shown in previous experiments (**Table 2**). The *18230C* mutant, when confronted with regular DL mutants in heterozygous females, yields a lethality that neither Dp1 nor Dp2 can rescue. Actually, the large Dp3 yields a low rate of escapers only. By contrast, Dp3 does rescue *18230C* when heterozygous over a RL mutant. These data prove that the DL, RL and *18230C* mutants affect the same function(s) although, most likely, to a different degree. Also, the incomplete rescue by the duplications is consistent with their heterogeneous transcriptional yield shown above (**Fig 4**).

To determine that *18230C* affects transcription of white isoforms, we carried out two experiments. On the one hand, adult females heterozygous for *18230C* and *hdp*^*3*^, whose dominant lethality is rescued by Dp2, show the classical wings up phenotype (**Table 3**). On the other hand, the qRT-PCR assay of these flies (genotype: *18230C/hdp*^*3*^*; Dp2/+*) shows depletion of exon 6d containing isoforms, C, F, G and H; confirming that *18230C* eliminates these white isoforms (**Fig 7**). Choosing Dp2 for these experiments is justified because it is the only available duplication that does not cover *hdp*^*3*^ and, yet, provides enough normal *wupA* function as to rescue the dominant lethality. The quantitative transcriptional levels provided by Dp2 are consistent with the unusual dosage effects observed in the rescue experiments (**Table 2**). Either one or two copies of Dp2 fail to yield normal levels of the 6d revealed isoforms, C, F, G and H. Although it shows a clear dosage effect, two copies of Dp2 still transcribe below 50% of controls.

The data from the other allele, *18230B*, were identical to those of *18230C*. Targeting the black ATG site would have not been instructive because, being located downstream of the white ATG, the procedure to obtain mutations by CRISPR in this triplet would also affect the white isoforms; thus providing no additional information with respect to the other existing DL rearrangement mutants.

**Fig 8:**
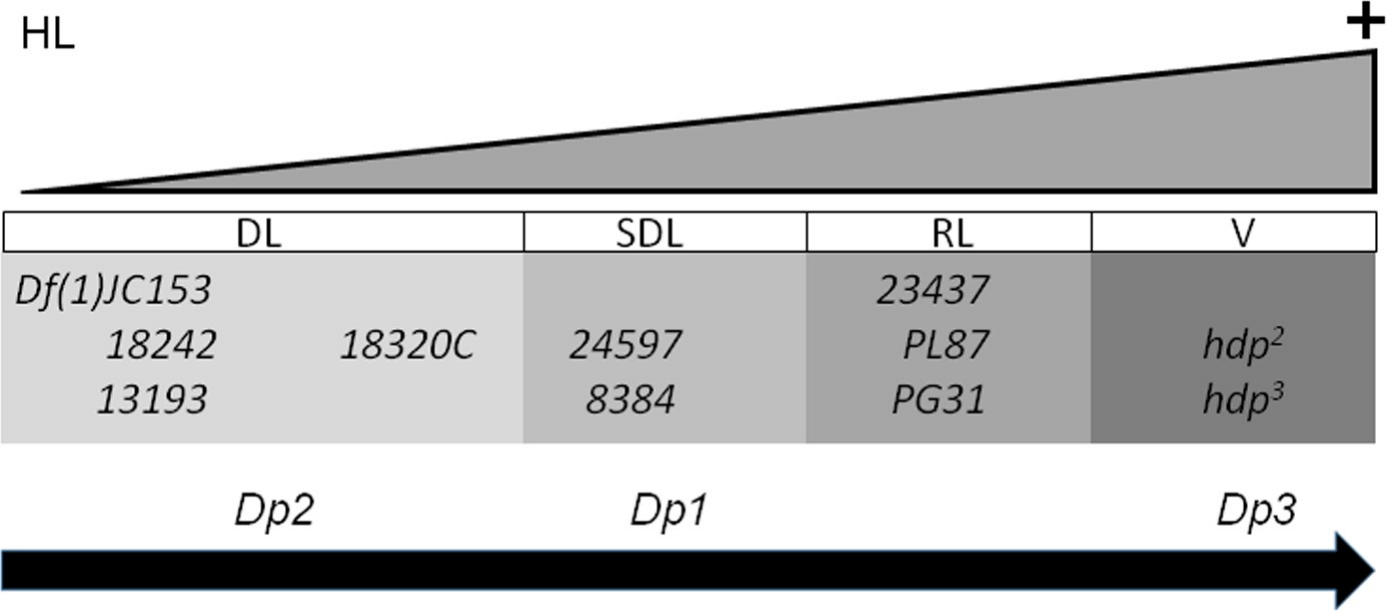
Graded scale of *wupA* mutant alleles. Schematic representation of the relative levels of *wupA* function in the corresponding mutant alleles and the duplications used in this study.

These data demonstrate that the elimination of the whole white subset of TnI isoforms is sufficient to cause a DL phenotype; thus, it seems that the HL function at 16F7 results from the haploinsufficiency of TnI proteins, at least those encoded by the white transcripts. Considering all available *wupA* mutants and genomic duplications studied here, a graded array of product depletion becomes evident (**Fig 8**). The most extreme is, obviously, the chromosomal deficiencies that uncover the region, and the DL rearrangements clustered at the 3’ end of the transcription unit. Following are the two *18230* mutants which also result in dominant lethality by eliminating the white isoforms only. The evidence that indicates that *18230* mutants are less severe than the regular DL mutants is shown in **Table 3**, where Dp3 can produce some escapers of the *18230C*/DL genotype [♀*18230C/18242*^*DL*^*; Dp3/+* or ♀*18230B*/*18242*^*DL*^*; Dp3/+* or ♀*18230C/13193*^*DL*^*; Dp3/+*]. This Dp3, in one dose only, does not rescue DL/DL genotypes at all. Although not analyzed here, the next grade of depletion would be the SDL type of mutants, followed by the RL type. Finally, the *hdp*^*3*^ mutation eliminates the exon 6d containing isoforms only, C, F, G and H. Their depletion, however, is not enough to cause lethality. Concerning the genomic duplications, a similar graded array of normal functions can be identified. The large Dp3 is the most effective supplier of all normal functions, followed, in descending order, by Dp1 and Dp2. Although the three duplications can rescue all DL and *wupA* mutants in general, Dp2 does not rescue *hdp*^*3*^ nor *hdp*^*2*^ alleles. The graded scale for *wupA* is equivalent to that observed for *dpp* alleles [47] (see **S7 Fig**). See Discussion for other functional similarities between *wupA* and *dpp*.

In conclusion, the *wupA* gene encodes a number of TnI protein isoforms which show functional diversity and interactions. Their transcriptional expression is quantitatively regulated to the extent that their combined haploinsuficiency, at least all the white isoforms, causes haplolethality.

## Discussion

Two major findings are reported here. A) TnI isoforms are functionally diverse and interacting, and B) This isoform repertoire is strongly dosage sensitive for organism viability to the extent of causing haplolethality.

### Biology of TnI isoforms

TnI was once considered a muscle specific protein playing its role within the Troponin-Tropomyosin complex during contraction of the sarcomere [48]. Our previous studies demonstrated that TnI is also expressed in non-muscle cells and that, at least some protein isoforms, play a role in normal and tumorous cell proliferation and in apico-basal cell polarity [33,39]. Here, we analyzed the TnI isoforms separately, and found that their role in cell proliferation is diverse and, in some cases, opposed. This implies a wide network of functional interactions between TnI isoforms themselves and with other partners. When the coding sequence of *wupA* was first identified [29], the array of alternative and mutually exclusive set of exons 6 a-d pointed to the possibility of functional specialization of TnI isoforms. One of the structural differences that the four alternative exons 6 offer is the number of Cys residues in each isoform. Differences in this number could provide different interactions with other Cys containing partners. This possibility should be explored in future protein-protein interaction studies using single TnI isoforms as bait.

We had shown previously that, at least some, TnI isoforms can translocate to the cell nucleus [32] where they elicit transcriptional changes in cell proliferation related genes [33]. Here we show that isoform K, and presumably the sequence identical L and M isoforms, can translocate to the nucleus. The high levels of Actin in the cell nucleus and its role in chromosome motion and gene transcription [49–51] is consistent with the proposal that the transcriptional changes elicited by the nuclear TnI could be mediated by the interaction of Actin with one or several TnI isoforms, akin to the interactions that take place in the muscle sarcomere. In the context of this study, at least isoforms A and B interfere with the nuclear trafficking of K. This feature provides a putative mechanism to influence cell proliferation through the quantitative regulation of the ratio between white and black TnI isoforms according to the cell status or physiological requirements. Experiments to address this hypothesis should be designed under conditions that ensure the quantitative control of combinations of TnI isoforms. Such experiments, however, are beyond the scope of this study given the vast number of possible combinations.

Concerning the mutant effects on the *wupA* products, it could be proposed that the DL mutant rearrangements may yield truncated TnI isoforms which could cause extreme interference leading to dominant lethality. Actually, truncated TnI forms are associated to cardiac dysfunction in vertebrates. These, usually C-terminus, truncated forms result from proteolysis of native cardiac TnI, and serve as diagnosis of myocardial infarction or stunning [52,53]. One of the TnI cleaving enzymes corresponds to the ubiquitin ligase MuRF1 [54]. However, the trigger factor, hence the origin of the cardiac dysfunction, of this proteolysis remains unknown. One N-terminus truncated TnI fragment in mice is produced under normal physiology and it becomes upregulated under microgravity conditions, which suggests a role in cardiac muscle adaptation [55]. However, for *wupA* and its associated HL condition, several types of data render the proposal of DL mutants as deleterious truncated forms, unlikely. The most direct evidence against truncated *wupA* products is that they are not detected in Western blots of extracts from various genotypes which include RL, SDL and DL types of mutants, all of them rearrangements (**S8 Fig**). None of these genotypes yield immune-detected protein fragments smaller than TnI. In addition, although all DL mutants known hitherto are rearrangements clustered towards the 3’ end of *wupA*, in this study we have obtained two A>C mutant alleles in the white ATG site, *18320B* and *18320C*, which also result in DL type of mutants. The available experimental evidence suggests only one functional difference between the DL rearrangements and the DL point mutation in the white ATG; while the former could be nulls for all TnI isoforms, the latter are likely nulls for the white subset only. Admittedly, counterarguments could still be raised to defend the hypothetical truncated TnI products, for example their rapid degradation and, therefore, their difficulty to be detected in Western blots. Would that be the case, however, the short life of truncated products would imply an equally short lived poisonous effect. Thus, there is no evidence to support it at this time.

### Regulation of *wupA* expression

The functional diversity of TnI isoforms implies the existence of regulatory mechanisms for proper expression in the normal biology of the cell. Previous to this study, we had identified two regulatory regions, URE and IRE, located at the 5’ terminus of *wupA*, based on the criteria of *LacZ*-reported expression of selected genomic fragments [31]. That criteria, however, is informative on positive enhancers, but repressors are missed. Here, we used qRT-PCR to discriminate among the various gene transcripts. The two genomic duplications analyzed, Dp1 and Dp2, although they rescue the DL mutants, the quantitative levels of transcripts they supply is heterogeneous with respect to the normal condition. This feature illustrates how superficial the evaluation of gene activity can be, if it is solely based on the resulting phenotype, adult viability in this case. Although these genomic duplications can restore viability of DL mutants, their transcription is far from being wild type. It is evident that the quantitatively normal levels of transcription require the proper chromatin landscape to an extent beyond the limits usually defined by a transcription unit. Additional supporting evidences can be found scattered in the scientific literature on other genes and organisms. However, these facts are largely unattended when experimenting with genomic fragments or, exceedingly so, when handling genetic constructs engineered with alien promoters and enhancers. The fact that none of the single isoforms rescue any of the known *wupA* mutants strongly suggests that the corresponding normal functions are achieved by combinations, rather than individual, isoforms acting in a specific stoichiometry. This is conceptually relevant because it underscores the combinatorial role of isoforms from a single gene, a subject often not considered in most studies.

The proximity and nature of *mir-969*, *lnc45605* and *lnc45606* genes invites to consider their possible regulatory role on *wupA*, and their contribution to the HL condition. In the case of *mir-969*, the feasible experimental data have failed to provide the required evidence. As for *lnc45605* and *lnc45606* the suitable genetic tools are not yet available. Thus, the issue of the regulatory mechanisms for *wupA* by means of additional genes remains open. Nevertheless, based on the facts that the heterozygous deletion for *lnc45605* and *lnc45606*, *Df(1)BSC352*, does not cause DL, and that their duplication in *Dp(1*,*2L)CH322-61E02r* does not rescue DL mutants, we can draw the conservative conclusion that the putative regulatory role of these genes upon *wupA* is independent from haplolethality. Furthermore, the fact that the A>C mutants *18320B* and *18320C* cause DL phenotypes, demonstrates that dosage reduction of *wupA* is sufficient to explain the HL condition.

The standard nucleotide map of the upstream *wupA* region (Flybase) and the position of RL type mutants determined by Southern blots, can be used to estimate the distance between putative regulatory landmarks. Using the 5’ end initiation site of *wupA* as a reference (nucleotide 18,116,922), the proximal order and relative nucleotide positions of landmarks will be: *PL87* (-30), distal break of *Df(1)23437* (-100), *PG31* (-249), distal break of *Df(1)BSC352* (-545), proximal break of *Df(1)23437* (-2100) and 3’ end of *lnc45605* (-3624). The overlap between *Df(1)BSC352* and *Df(1)23437* explains their non-complementation for their recessive lethality. However, the same non-complementation with *PG31* and *PL87* may be attributed to polar effects during regional pairing caused by the different nature of the two rearrangements, deficiency and insertions, or to dysfunction of different sets of *cis*-acting regulatory regions in each chromosome. A regulatory role for one or both of the *lnc* genes, although it cannot be ruled out, seems unlikely. Over 3 Kb separate *lnc45605* from *wupA*, and the *Dp(1*,*2L)CH322-61E02r*, which contains both *lnc* genes, does not rescue *wupA* mutants of any type. The later feature prevents to call even for a *trans*-acting regulatory role of *lnc* genes on *wupA*.

The case of *hdp*^*3*^ has unveiled another unexpected feature on quantitative control of *wupA* transcription. A single nucleotide mutation at the splicing acceptor site for exon 6d increases the expression of non-exon 6d containing transcripts. These results on transcriptional activity could indicate that the expression of *wupA* is subject to a feed-back control by its own encoded products, in addition to the two ATG sites, and by the duplicated regulatory 5’ regions, URE and IRE. At this time, however, this form of *wupA* regulation must be considered hypothetical.

Attending to the clustering of several DL mutant rearrangements, a regulatory structure in the interval between coordinates 1086.8 and 1086.9 towards the 3’ end of the gene could be proposed. The region corresponds to the intron between exons 7 and 8. Of note, the rearrangements leading to DL mutations have occurred in non-coding sequences in all HL associated genes known to date in flies and mice. This putative regulatory structure in *wupA* cannot operate in *trans* since the combination of the genomic fragments *E6L* plus *Dp(1;2R) CH322-143G12r* does not rescue DL mutants. Also, the URE and IRE regulatory regions cannot be the sole mechanism that regulates *wupA* expression because their structural alteration by means of deletions (*l(1)23437*) or insertions (*PL87* and *PG31*), yield RL, but not DL, phenotypes. Nonetheless, the URE-IRE regions exhibit chromosome pairing effects whereby the homozygosis for *PL87* restores normal expression of the gene [31]. Although the *LacZ* reported expression domains instructed by URE and IRE seem identical, they are not functionally redundant because the homozygosis for *l(1)23437*, which deletes the URE domain only, is still lethal [31]. Likewise, the homozygosis for any of the DL rearrangements maintains the dominant lethality. The clustering of DL rearrangements towards the 3’ end of *wupA*, plus the failure of incomplete genomic fragments to rescue DL phenotypes, suggests that the integrity of the transcription unit and, likely, some adjacent sequences upwards of its 5’ end, is required for the normal expression of the gene. This clustering of DL mutants would be akin to regulatory landscapes proposed for some developmental genes [56]. Consistent with this proposal, the available database information on Hi-C domains [57,58] indicate that the *wupA* gene is contained within a single tridimensional chromatin domain (http://epigenomegateway.wustl.edu/browser). Thus, the native chromatin in and around *wupA* could be required to support the hypothetical structural regulatory component of its HL function. The role of chromatin structure on the transcriptional regulation of adjacent genes, however, is still a matter of debate including the potential tissue dependent differences of Hi-C domains [59]. Quantitative regulation of gene transcripts can be achieved by a number of mechanisms, including their codon identity composition, which affects their stability [60], or through modified tRNAs, which affect their translational efficiency [61]. Also, genomes may differ in their DNA content, both between cells and between individuals, and this variation is thought to contribute to adaptation and evolution [62–65].

Although the actual mechanism for *wupA* regulation is still unknown, the existing data underscore the fact that that mechanism must ensure a proper quantitative control among TnI isoforms for normal biology. Is this quantitative regulation a peculiarity of HL genes?

### Haplolethal versus Haploinsufficient genes

From yeast to flies, the haploid condition for genes encoding proteins with very general functions in cell biology results in haploinsufficiency. For instance, *Minute* genes in *Drosophila* are involved in translation or general metabolism, and their heterozygous *M/+* condition causes developmental delay, reduced size and low viability [66]. However, out of the 66 *Minutes* identified so far [21], none of them can be linked to a HL gene. Actually, none of the known mutants in *wupA* or *dpp* show *Minute*-like phenotypes. Another example is provided by the RNA-polymerase II subunits encoding genes. Out of the 13 members of this family [67,68], none of them have been linked to HL regions. Equivalent to fly *Minutes*, the 170 haploinsufficient genes in yeast can be recovered as viable organisms if the culture medium is metabolically adjusted [5]. Thus, a basic biological role of the encoded products does not seem to justify a HL condition (see also Tabl4 in [21] for other haploinsufficient, non haplolethal, genes).

The location of *wupA* on the sexually dimorphic X chromosome invited to consider the possibility that its HL condition could result from a defective form of dosage compensation mechanisms akin to those already known to tune up the transcription of X-linked genes in males [69–72]. However, our previous experiments addressing the possibility of altered dosage compensation for *wupA* mutants yielded negative results [26]. Also, *dpp* and two other HL cases are located in autosomes. The relevant question, then, is what is so peculiar about HL genes?

Now that two HL genes, *dpp* and *wupA*, are known in some depth we can try to point out similarities among them. Two features are in common in both cases, the corresponding DL mutants are chromosomal rearrangements, and the affected gene encodes several transcripts. These two features can hardly be considered as distinguishing peculiarities with respect to other genes. It could be argued that one or several gene products are required in a critical amount for viability. The case of *wupA* HL shows (**Table 2**) that none of the transcriptional products, taken one at a time, rescue the DL mutants; thus, it is clear that the hypothetically critical amount for viability should correspond to more than one product. We co-overexpressed several of the UAS constructs (see foot note on **Table 2**) but none of them rescued the DL phenotype. Only when all white subset of *wupA* products are eliminated (mutants *18320C* and *18320B*), a DL phenotype is obtained. Thus, the DL mutant phenotype seems to result from the cumulative depletion of many TnI isoforms, which play diverse roles in muscle contraction, apico/basal cell polarity, cell proliferation, gene transcription, among others. It is worth pointing out that TnI is an Actin-binding type of protein and, consequently, its presence would be required in most force generating mechanisms in the cell. We envision that the functional diversity among the 11 *wupA* proteins represents a wide repertoire of mechanistic specificities, and the DL mutants are, in effect, a combined haploinsufficiency for the entire, or most of it, repertoire. As for *dpp*, although only one protein is known, DPP, its acting transcription factor is the *Mothers-against-dpp* (MAD) complex which includes the CDK8 and CycC components [73], a feature that seems akin to the role of TnI upon CDK2 and CDK4. In both cases, these signaling pathways control cell proliferation and several other cell signaling processes.

While the still scant information about HL genes of *Drosophila* is increased, it may be of interest to inquire about their homologues in vertebrates. The fly genes *wupA* and *dpp* have homologues in humans, *TNNI* and *BMP*, respectively. However, neither of them has maintained their HL condition in vertebrates. While *wupA* encodes 11 TnI isoforms, the TNNI human counterparts are encoded in three different genes, *TNNI1*, *TNNI2* and *TNNI3*. Based on the association between copy number variation and certain types of cancer (Catalogue of Somatic Mutations in Cancer: https://cancer.sanger.ac.uk/cosmic), and experimental tumor cell growth suppression, *TNNI1* seems the closest homologue to *wupA* [18]. The sequence similarity between the three human genes versus the single fly gen, however, is not very different (31, 32 and 34%, respectively). The corresponding chromosomal locations, 1q32.1, 11p15.5 and 19q13.42, respectively, are not included in the regions never recovered in haploid condition. Nevertheless, whole genome analysis of the predicted probability of being haploinsufficient, indicates that *TNNI3* has a high probability [1]. A protein interactome data base (http://www.interactome-atlas.org/search) shows that the three human TNNI interact with several proteins beyond the classical components of the muscle sarcomere. Notably, TNNI1 exhibits the largest repertoire of interactions including, among others, RPAC1, a DNA-dependent RNA polymerase, and CDC7AL, a cell cycle associated protein. These features are consistent with the transcriptional and cell proliferation effects identified here for *Drosophila* TnI. As for DPP/BMP, although the fly gene encodes 1 protein through 5 transcripts, the human homologue is represented by a family of 10 members, from which *BMP2* and *BMP4* can be considered the closest to *dpp*. Concerning *Vegf* and *Tcof1*, the murine and human homologs have in common the coding for several proteins, and the presence of multiple (*Vegf*) or two (*Tcof1*) ATG sites (www.Ensembl.org).

One would have expected that HL functions will be under such a negative selective pressure as to have been eliminated. Yet, they seem to exist in far apart species; *Drosophila* contains five of them, and mice at least two. Actually, this paradox extends to haploinsufficient genes since they represent a barrier to organismal fitness due to the resulting pathologies. A dosage-stabilizing hypothesis has been proposed to explain the persistence of haploinsufficient genes, based on the possibility that, both in their attenuated and excess of function conditions are deleterious for the organism [64]. Although we do not argue against this proposal, we find it unlikely for haplolethality because large duplications covering each of these fly HL regions, except Tpl, are viable. We speculate that HL functions have been maintained in evolution because they exhibit two key features: 1) The multiplicative effect of dosage reduction of several functionally related products, and 2) The complex regulatory mechanism among these products which need to be expressed in specific quantities and combinations. Since all fly TnI isoforms are encoded in a single gene, its HL condition becomes unavoidable. Only when the repertoire of products splits into separate genes, as after gene duplication, the initial HL function could dissociate from the initial single gene. That dissociation, however, would be apparent only. Likely, the combined haploidy of the duplicated genes would reveal again the original haplolethality. Under this hypothesis, haplolethality would not be a property of the gene, but a property of the quantitative requirements of the ensemble of encoded products. A prediction of this hypothesis will be that the combined haploinsufficiency of the three *TNNI* genes of vertebrates should result in a DL phenotype. Also, it can be expected that functional interactions or interferences among vertebrate TNNI isoforms are likely to occur. A similar prediction could be made for the combined haploidy of most, if not all, BMP genes of vertebrates. A systematic search for HL functions in a particular genome could unveil regulatory interactions unsuspected hitherto. In all likelihood, the HL condition of *wupA* in *Drosophila* is not unique across genomes. However, to find out if other HL regions result from a quantitative regulation of combinations of gene products, equivalent studies to those carried out here will be necessary. The data reported here underscore the biological relevance of protein dosage of functionally related products.

## Materials and methods

### Mutant strains

Flies were raised in standard fly food at 25°C. As *wupA* mutations we used the following fly stocks from our own collection: *Df(1) TnI*^*23437*^, *In(1)PL87* and *In(1)PG31* [33] are three rearrangements located the 5`URE and IRE regulatory region of the gene [31] (see also **Fig 2**). Other mutant alleles and genomic constructs have been described previously [26,30]. To elicit excess of *wupA* function, we used the *PBac(WH)f06492* construct from Exelixis referred here as *UAS-TnI*^*f06492*^. The genomic fragments *CH322-143G12* (22251 bp), *CH322-61E02r* (20843 bp) or *CH322-04P16r* (18542 bp) were generated in [74]. The three fragments were cloned in the vector attB-P[acman]-CmR-BW-F-2-attB-BW3 (accession FJ931533). Each bacterial artificial chromosome (BAC) was injected in embryos of the stock *y*^1^ *M{vas-int*.Dm}ZH-2A w[*]; M{3xP3-RFP.*attP’}ZH-22A* (BL24481) and adult transgenic flies were selected by the reporter RFP signal in the oceli. To analyze other HL regions, the following stocks were obtained from the Bloomington collection: *C(X;Y)*, *y sn Grip91/C(1)RM*, *y v; Dp(1;f)LJ9*, *y*^*+*^ (BL5128); *Dp(2;1)G146*,*dpp*^*+*^*/FM7i; dpp*^*H46*^ *wg*^*Sp*^ *cn bw/CyO* (BL2060); *Df(1)hl-a*, *w cv B/FM6; Dp(1;2)sn*^*+72d*^*/+* (BL6698) and *Dp(1;2)CH322-143G12r/CyO; UAS(y*^*+*^*v*^*+*^*) up*^*RNAi*^*attP2/TM6* (from BL31541).

### Generation of UAS-*wupA* transgenic lines

All DNA plasmids were generated by RECOMBINA S.L. (Navarra, Spain). Full length DNA sequence of each *wupA* transcript was amplified by PCR. The products were cloned in pENTRY vector as an intermediate step. Then, each *wupA* fragment was subcloned in pUASp vector via NotI/XbaI restriction enzymes and injected in *y w* embryos. For the HA-tagged version of the K isoform, the *wupA-K* coding sequence was amplified by PCR. The primers *wupA-K-HA* forward and *wupA-K-HA* reverse introduced an in-frame HA epitope coding sequence in 3´. The fragments were cloned in pENTRY vector and subcloned in pUASp vector via KpnI/XbaI restriction enzymes and injected in *y w* embryos. Sequence primers were as follows:

*wupA-K-HA* F>cggGGTACCatggaggaagcctccaaggccaa
*wupA-K-HA R*>tagTCTAGAttaAGCGTAATCTGGTACGTCGTATGGGTAagcttcggcctcaacctcct

### Somatic clone induction and quantification

Crosses were set with 10 females and 10 males per vial at 25°C changed every 72 hours to avoid overcrowding. FLP-out clones were obtained by delivering a heat shock (8 minutes at 37°C) during 2^nd^ instar larvae of *HsFlp; Act-FRT-yellow-FRT-Gal4; +* crossed against the corresponding UAS-*wupA* transgenic line isoform (A, -B, -C, -D, -E, -F, -G, -H, -I, -J or -K). 48 hours after heat shock, 3^rd^ instar wandering larvae were dissected for clone screening. Control cultures (UAS-*LacZ*) were run in parallel. A software-assisted area measurement (Bitplane’s Imaris Surface) was used to obtain clone area and cell size. Cell profiles are identified by the myrRFP reporter and cell nuclei are revealed by DAPI. The “surface” option was chosen to measure the areas occupied by DAPI or RFP pixels from the entire 3D image. The area of the clone (RFP-marked) was calculated and divided by DAPI-marked area. As a result, the percentage of wing disc occupied by genetically marked cells was represented.

### Viability quantification

To determine the rescue of dominant lethal phenotypes, we performed viability assays. 10 females and 10 males were crossed and maintained in the same tube for 72 hours, then adult flies were changed to a new tube and embryos were incubated at 25°C. The total number of adult flies was counted in three independent experiments. The number of experimental adult flies was divided by the number of control siblings (balancer) adult flies to indicate the survival ratio.

### Quantitative PCR assays

For qRT-PCR assays, RNA was extracted with Trizol (Invitrogen) according to standard procedures. To prevent genomic DNA contamination all RNA samples were treated with DNaseI according to manufacturer´s procedures (30 min at 37°C). Primers were designed to anneal in different exons from each gene, later qPCR products were run in 2% agarose gels to analyze the bands. All the products correspond with their expected length, which indicates that no genomic DNA contamination was present. Assays were performed in triplicates using RNApolII as a housekeeping gene. Drosophila Troponin-I TaqMan Gene Expression probe (Applied Biosystems) was used. Used primers were:

wupA 5-6F GGCTAAACAGGCTGAGATCG
wupA 6a R TCGATGATGCGTCTACGTTC
wupA 6b R TCAACATCCTTGCGTTTAACA
wupA 6c R AAATCGTACTTTTCGGACTCCA
wupA 6d R AGATCCCATTTCTGGCCTTC
wupA 11/12 F GCCCAAGTTAACGATCTTCG
wupA 11/12 R TCCAGCGTGAACTCCTTCTT
RpL32 F TGTCCTTCCAGCTTCAAGAT
RpL32 R CTTGGGCTTGCGCCATTTG

### Statistics

Statistical significance was calculated with the two-tailed Student’s *t*-test or ANOVA test. Significance levels are indicated as *p < 0,05; **p < 0,005 or ***p < 0,001. Number of samples N > 8 animals in confocal imaging, and n = 3 sets of 5 adults, male or female as indicated, for qPCR experiments.

## Supporting information

S1 Fig Physical map of *Df(1)BSC352* with respect to *wupA* and adjacent proximal genes. Data are from FlyBase. The deficiency, highlighted in yellow, is heterozygous viable although it deletes the three proximal genes *lnc45605*, *lnc45606* and *mir-969*.
(TIF)

S2 Fig Functional analysis of *Df(1)BSC352*. Higher magnification of the coordinates map from FlyBase shown in the previous figure, focused on the region between the 5’ end of *wupA* transcription unit and the distal end of *Df(1)BSC352*. Note that the extent of *Df(1)23437* is based on Southern data and, consequently, its precise nucleotide coordinates are not known. Although this deficiency is unlikely to delete part of *lnc45605*, it could perturb its transcription due to a polar effect (see main text). *Df(1)BSC352* is lethal over *PL87*, *PG31* or *Df(1)23437*, all lethal rearrangements affecting the regulatory URE region of *wupA*. This lethality, however, is rescued by Dp1, Dp2 or Dp3, duplications which include *lnc45605* and *lnc45606* (see main text). By contrast, *Df(1)BSC352* does complement the wing position phenotype of *hdp*^*2*^ and *hdp*^*3*^. These data are compatible with the existence of regulatory sequences upstream of the 5’ end of *wupA*, possibly including the *lnc45605* and/or *lnc45606* genes. This regulatory activity, however, is not the sole regulator of *wupA* expression since *Df(1)BSC352* is not a dominant lethal.
(TIF)

S3 Fig Transcriptional effects of TnI isoform K overexpression upon selected genes. Set of genes tested in the qRT-PCR assays of *tub-Gal4*^*LL7*^*>UAS-TnI-K* female larvae using *tub-Gal4*^*LL7*^*>UAS-LacZ* female larvae from a parallel cross as control. All assay determinations were done in triplicate.
(TIF)

S4 Fig Overexpression of TnI isoform K in wing domains cause morphological abnormalities. Adult wings expressing isoform K in the *rn-Gal4* domain. Note the curved wings (arrows). These adults exhibited low viability with respect to siblings.
(TIF)

S5 Fig Transcriptional effects of TnI isoform K overexpression upon other TnI isoforms. Primers from exons 6 were used to discriminate among TnI isoforms in these qRT-PCR assays, and a HA-tagged isoform K was used for overexpression in *tub-Gal4*^*LL7*^ *> UAS-HA-TnI*^*K*^ larvae. Note the rather selective effect on 6a revealed isoforms which include isoform K.
(TIF)

S6 Fig Design of CRISPR/Cas9 mutations in the white ATG site of *wupA*. Mutations *18320B* and *18320C* were produced by WellGenetics Inc. (Taiwan). The later was validated by sequencing.
(TIF)

S7 Fig Schematic representation of *dpp* allelic series. The graded allelic series of increasing severity is reported in [47].
(TIF)

S8 Fig Western blots of DL, SDL and RL mutants of *wupA*. Adult protein extracts from various genotypes were hybridized against J4 anti-TnI [32] and anti-Tubulin antibodies. The relative intensity of the TnI band is consistent with the transcriptional effects of Dp1 and Dp2 shown in Fig 4. In addition, note the absence of protein bands with molecular weights bellow TnI, which argues against the presence of detectable truncated TnI products.
(TIF)

S1 Table Genotypes tested in the functional analysis of Dp1 and Dp2 (see Table 1) and in the HL regions interaction assay. (DOCX)
