# Supplementary figures and images for: The haplolethality paradox of the wupA gene in Drosophila
Source: PLoS Genet. 2021 Mar 19;17(3):e1009108. doi: 10.1371/journal.pgen.1009108 (PMC8011728; doi:10.1371/journal.pgen.1009108)

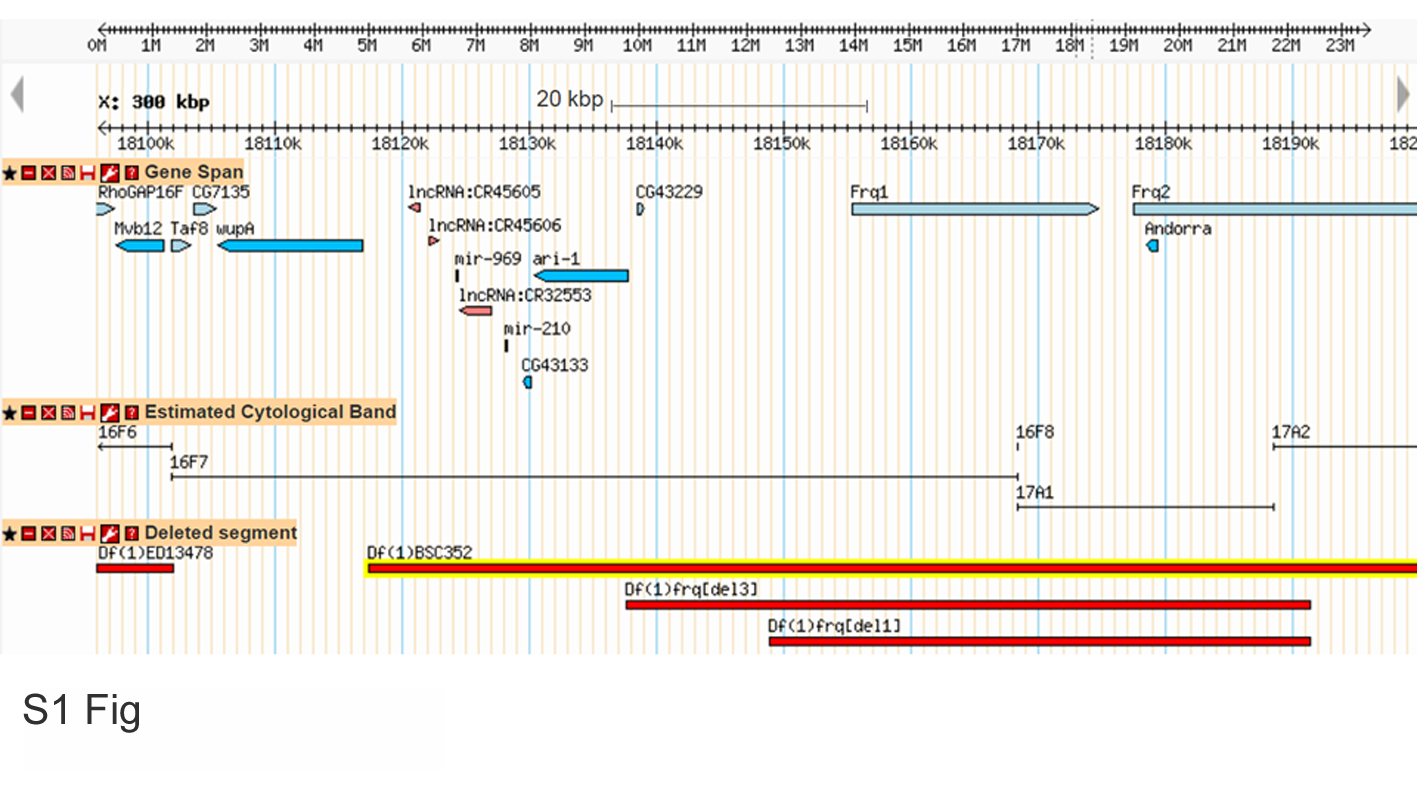

Supplement: S1 Fig — Data are from FlyBase. The deficiency, highlighted in yellow, is heterozygous viable although it deletes the three proximal genes lnc45605, lnc45606 and mir-969. (TIF) [file pgen.1009108.s001.tif]

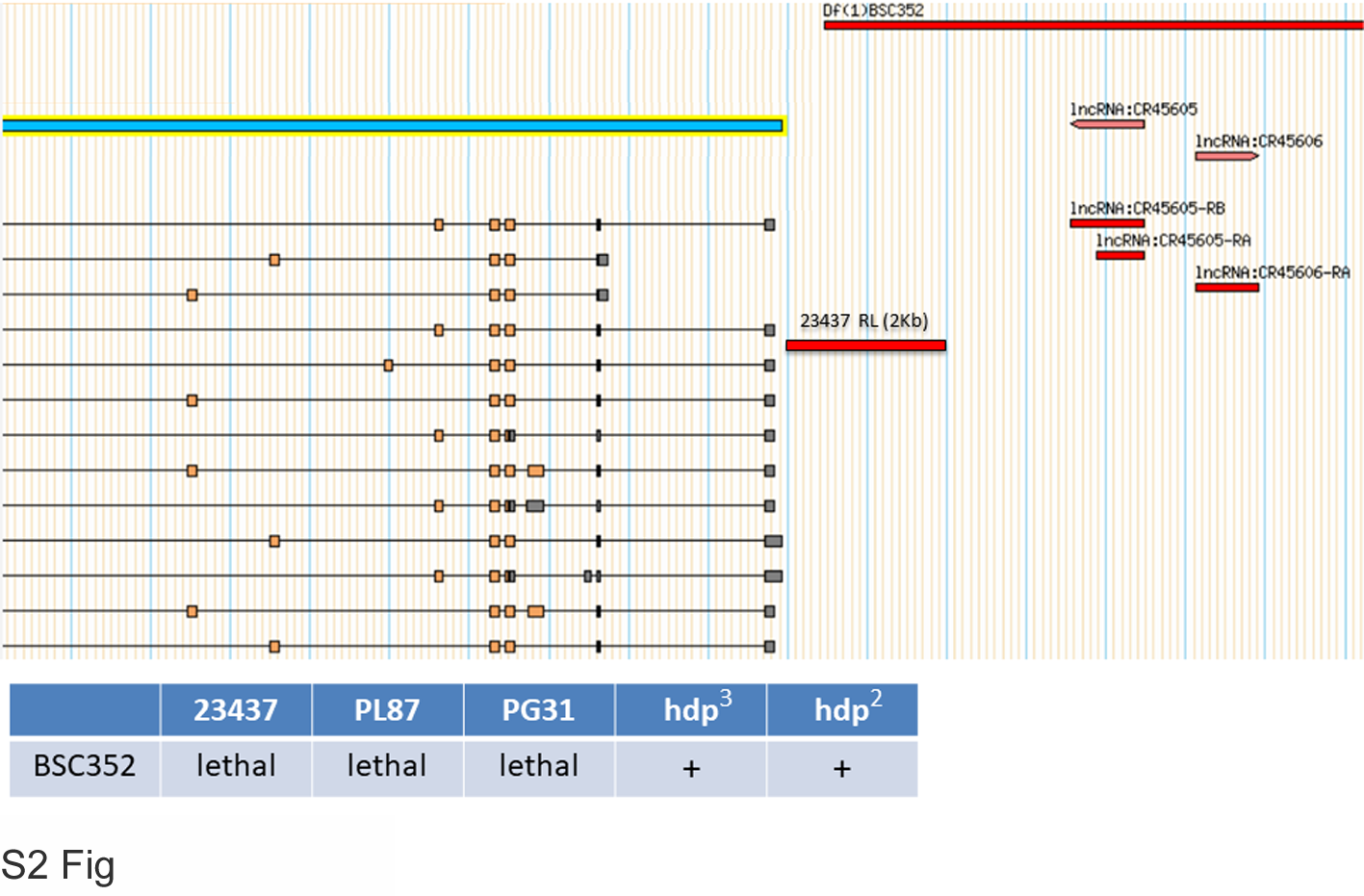

Supplement: S2 Fig — Higher magnification of the coordinates map from FlyBase shown in the previous figure, focused on the region between the 5’ end of wupA transcription unit and the distal end of Df(1)BSC352. Note that the extent of Df(1)23437 is based on Southern data and, consequently, its precise nucleotide coordinates are not known. Although this deficiency is unlikely to delete part of lnc45605, it could perturb its transcription due to a polar effect (see main text). Df(1)BSC352 is lethal over PL87, PG31 or Df(1)23437, all lethal rearrangements affecting the regulatory URE region of wupA. This lethality, however, is rescued by Dp1, Dp2 or Dp3, duplications which include lnc45605 and lnc45606 (see main text). By contrast, Df(1)BSC352 does complement the wing position phenotype of hdp2 and hdp3. These data are compatible with the existence of regulatory sequences upstream of the 5’ end of wupA, possibly including the lnc45605 and/or lnc45606 genes. This regulatory activity, however, is not the sole regulator of wupA expression since Df(1)BSC352 is not a dominant lethal. (TIF) [file pgen.1009108.s002.tif]

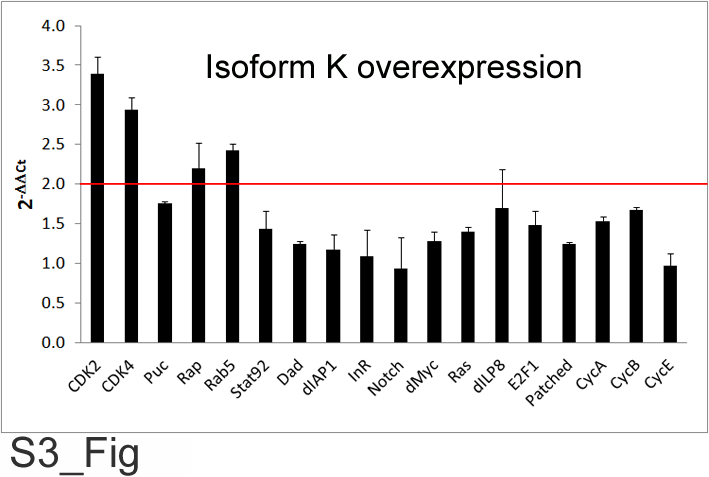

Supplement: S3 Fig — Set of genes tested in the qRT-PCR assays of tub-Gal4LL7>UAS-TnI-K female larvae using tub-Gal4LL7>UAS-LacZ female larvae from a parallel cross as control. All assay determinations were done in triplicate. (TIF) [file pgen.1009108.s003.tif]

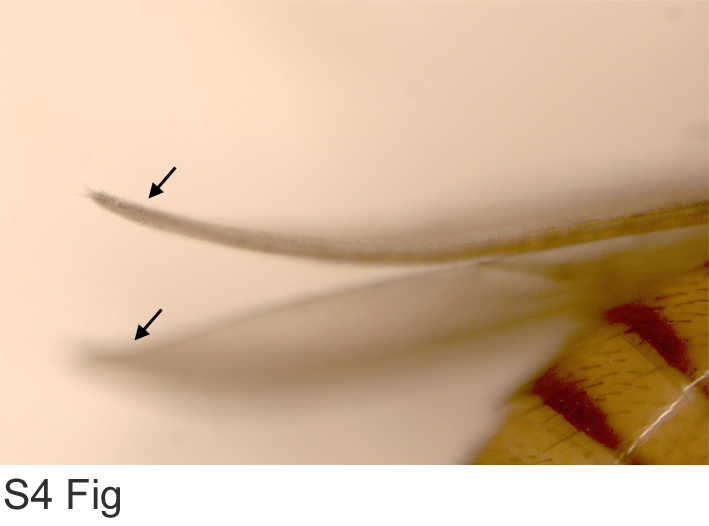

Supplement: S4 Fig — Adult wings expressing isoform K in the rn-Gal4 domain. Note the curved wings (arrows). These adults exhibited low viability with respect to siblings. (TIF) [file pgen.1009108.s004.tif]

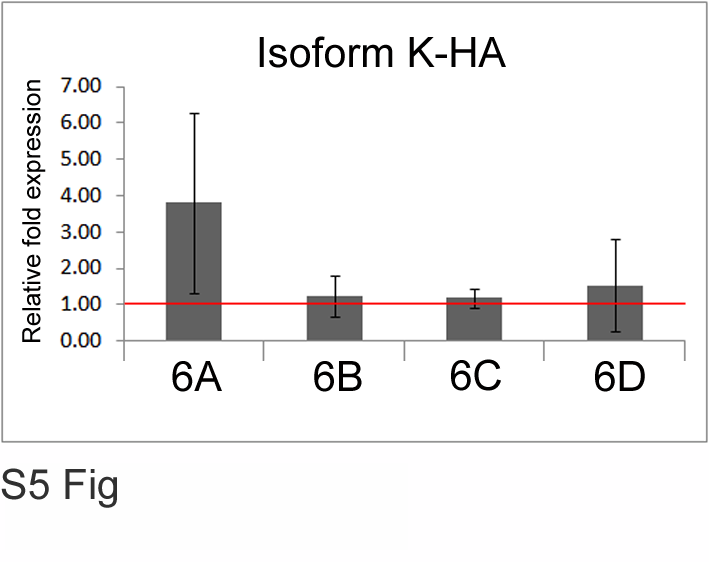

Supplement: S5 Fig — Primers from exons 6 were used to discriminate among TnI isoforms in these qRT-PCR assays, and a HA-tagged isoform K was used for overexpression in tub-Gal4LL7 > UAS-HA-TnIK larvae. Note the rather selective effect on 6a revealed isoforms which include isoform K. (TIF) [file pgen.1009108.s005.tif]

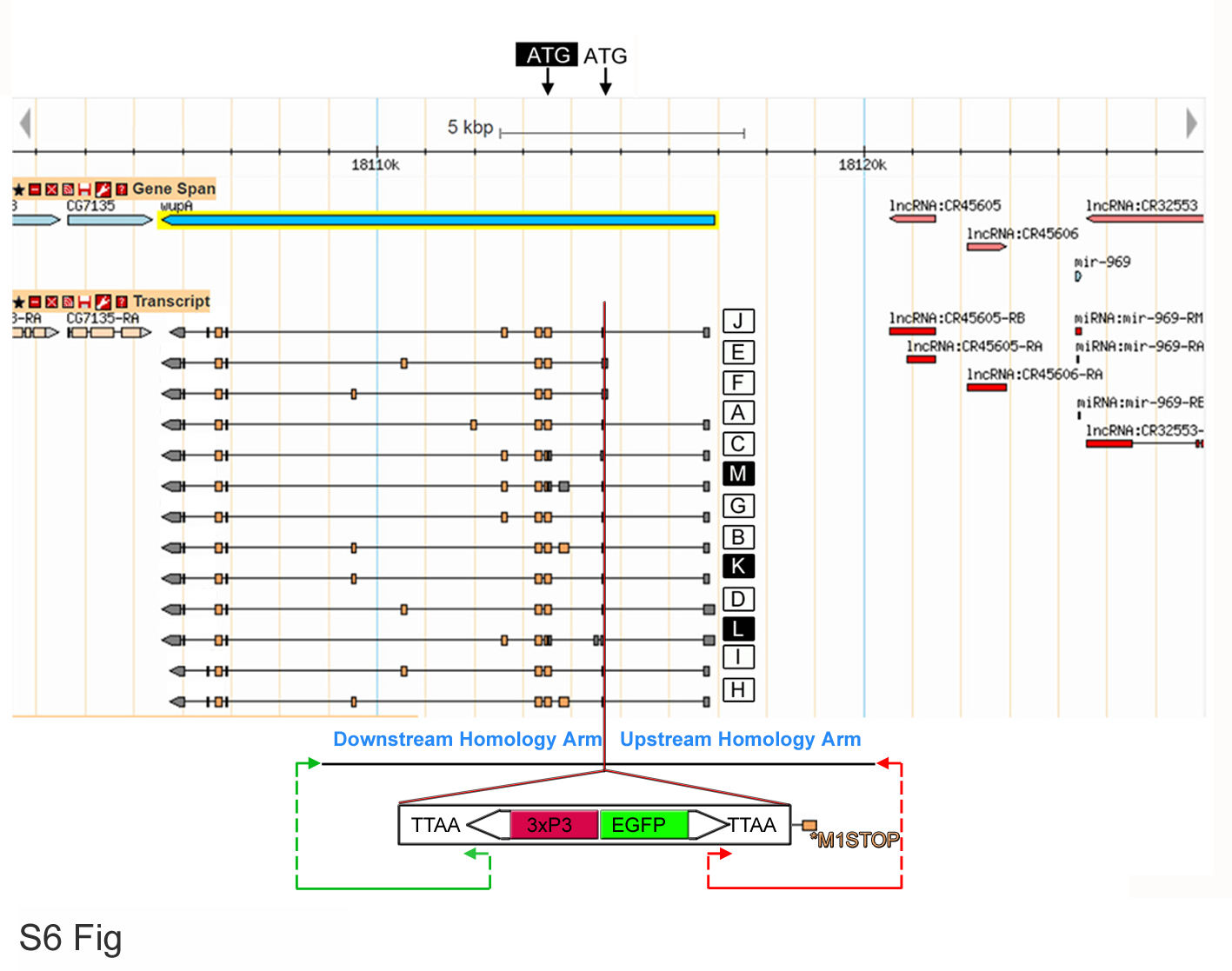

Supplement: S6 Fig — Mutations 18320B and 18320C were produced by WellGenetics Inc. (Taiwan). The later was validated by sequencing. (TIF) [file pgen.1009108.s006.tif]

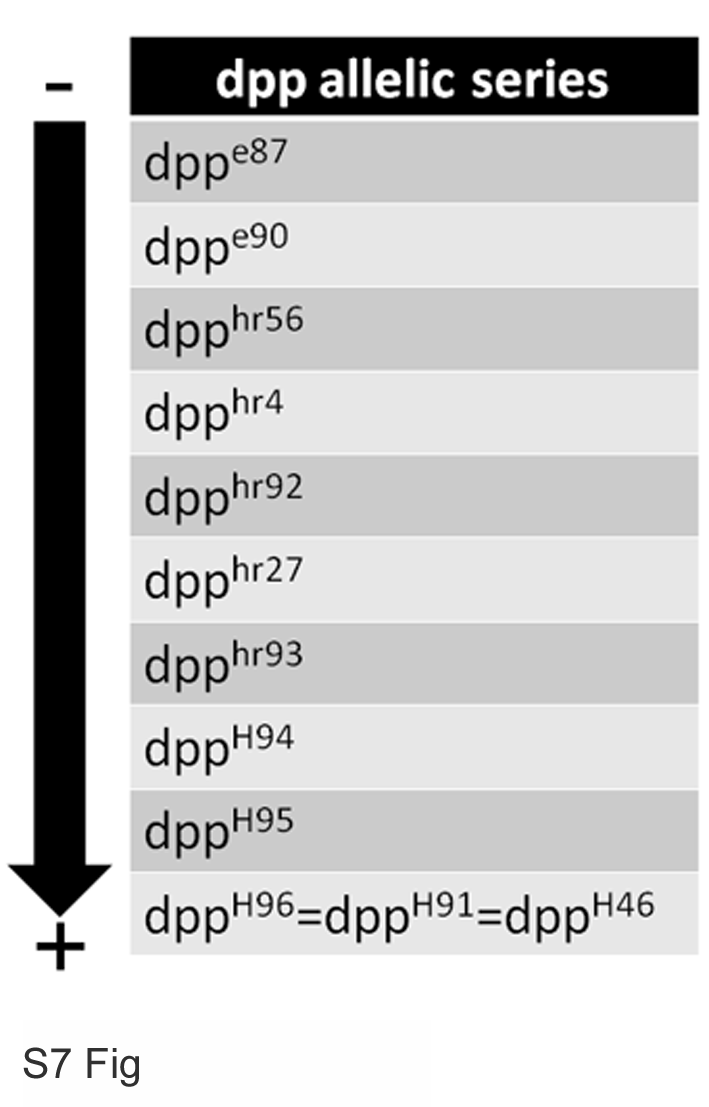

Supplement: S7 Fig — The graded allelic series of increasing severity is reported in [47]. (TIF) [file pgen.1009108.s007.tif]

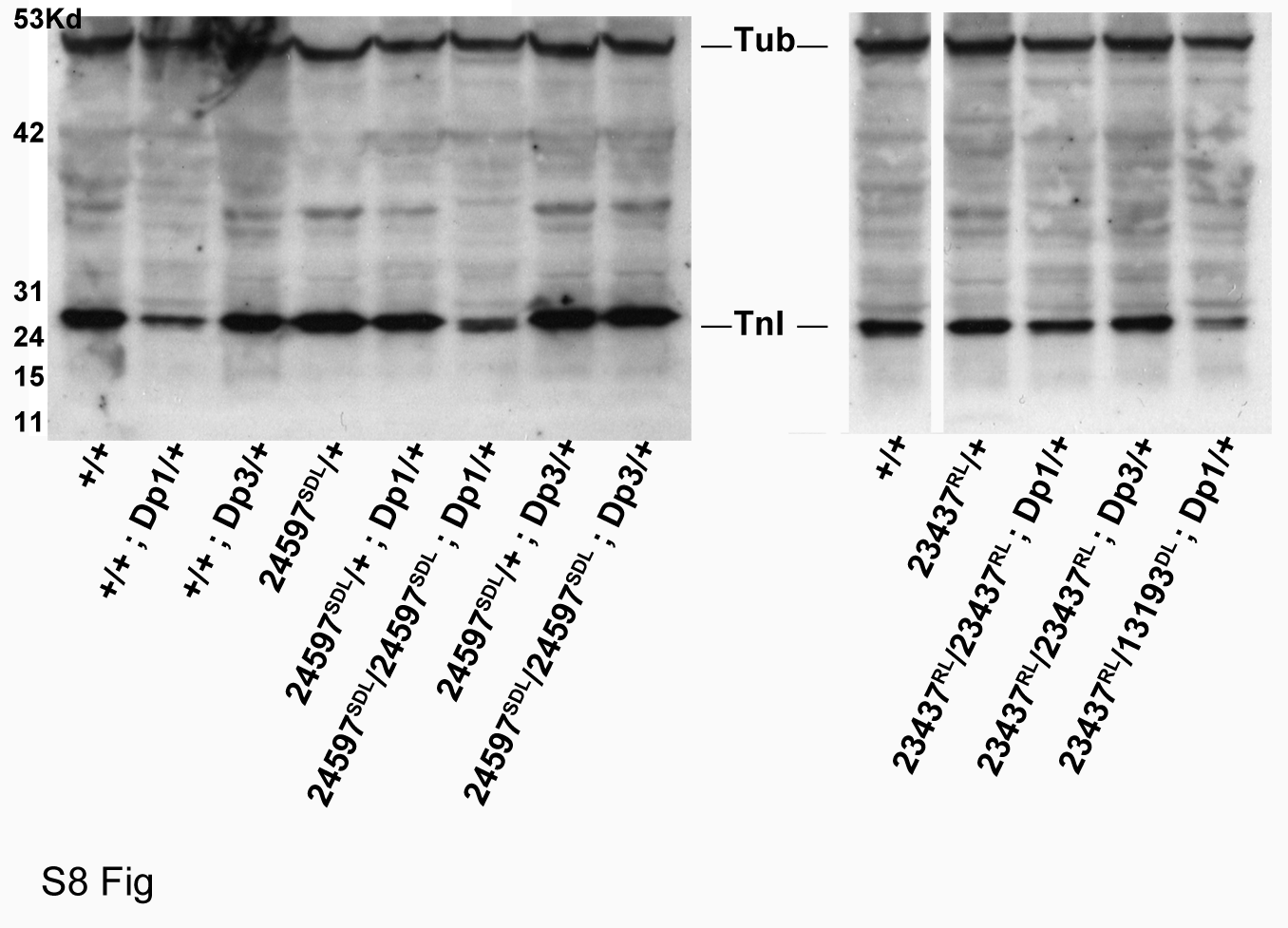

Supplement: S8 Fig — Adult protein extracts from various genotypes were hybridized against J4 anti-TnI [32] and anti-Tubulin antibodies. The relative intensity of the TnI band is consistent with the transcriptional effects of Dp1 and Dp2 shown in Fig 4. In addition, note the absence of protein bands with molecular weights bellow TnI, which argues against the presence of detectable truncated TnI products. (TIF) [file pgen.1009108.s008.tif]
