# Supplementary material for: The haplolethality paradox of the wupA gene in Drosophila
Source: PLoS Genet. 2021 Mar 19;17(3):e1009108. doi: 10.1371/journal.pgen.1009108 (PMC8011728; doi:10.1371/journal.pgen.1009108)
Supplement: S1 Table — (DOCX) [file pgen.1009108.s009.docx]

**S1 Table.** Genotypes tested in the functional analysis of Dp1 and Dp2 (see **Table 1**) and in the HL regions interaction assay

| **Genotype** | **Viability /Phenotype** |
| --- | --- |
| **Data of Table 1** |  |
| ♂ *f^5^* *l(1)18242^DL^ os*; *Dp(1;2R) CH322-143G12r* /+ | √ |
| ♂ *y w* *l(1)13193^DL^ os*; *Dp(1;2R) CH322-143G12r* /+ | √ |
| ♀ *f^5^* *l(1)18242^DL^ os/+*; *Dp(1;2R) CH322-143G12r* /+ | √ |
| ♀ *y w* *l(1)13193^DL^ os/+*; *Dp(1;2R) CH322-143G12r* /+ | √ |
| ♀ *f^5^* *l(1)18242^DL^ os/ f^5^* *l(1)18242^DL^ os*; *Dp(1;2R) CH322-143G12r* /+ | **†** |
| ♀ *y w* *l(1)13193^DL^ os / y w* *l(1)13193^DL^ os*; *Dp(1;2R) CH322-143G12r* / *Dp(1;2R) CH322-143G12r* | <1% |
| ♂ *f^5^ l(1)23437^RL^ os ; Dp(1;2R) CH322-143G12r* /+ | √ |
| ♀ *f^5^ l(1)23437^RL^ os / f^5^ l(1)23437^RL^ os ; Dp(1;2R) CH322-143G12r* /+ | √ |
| ♀ *f^5^ l(1)23437^RL^ os / f^5^* *l(1)18242^DL^ os ; Dp(1;2R) CH322-143G12r* /+ | √ |
| ♂ *f^5^ hdp^2^ ; Dp(1;2R) CH322-143G12r* /+ | + |
| ♂ *y w* *hdp^3^; Dp(1;2R) CH322-143G12r* /+ | + |
| ♂ *f^5^* *l(1)18242^DL^ os* ; *wupA-2XTY1-sGFP-V5-preTEV-BLRP-3XFLAG* /+ | **†** |
| ♂ *y w* *l(1)13193^DL^ os* ; *wupA-2XTY1-sGFP-V5-preTEV-BLRP-3XFLAG* /+ | **†** |
| ♂ *f^5^* *l(1)18242^DL^ os*; *wupA-2XTY1-sGFP-V5-preTEV-BLRP-3XFLAG* / *wupA-2XTY1-sGFP-V5-preTEV-BLRP-3XFLAG* | √ |
| ♀ *f^5^* *l(1)18242^DL^ os /+* ; *wupA-2XTY1-sGFP-V5-preTEV-BLRP-3XFLAG* /+ | √ |
| ♀ *y w* *l(1)13193^DL^ os/+*; *wupA-2XTY1-sGFP-V5-preTEV-BLRP-3XFLAG* /+ | √ |
| ♀ *f^5^* *l(1)18242^DL^ os / f^5^* *l(1)18242^DL^ os* ; *wupA-2XTY1-sGFP-V5-preTEV-BLRP-3XFLAG* /+ | **†** |
| ♀ *y w* *l(1)13193^DL^ os/ y w* *l(1)13193^DL^ os*; *wupA-2XTY1-sGFP-V5-preTEV-BLRP-3XFLAG* /+ | **†** |
| ♀ *y w* *l(1)13193^DL^ os/ y w* *l(1)13193^DL^ os*; *wupA-2XTY1-sGFP-V5-preTEV-BLRP-3XFLAG* / *wupA-2XTY1-sGFP-V5-preTEV-BLRP-3XFLAG* | <1% |
| ♀ *f^5^* *l(1)18242^DL^ os / f^5^* *l(1)18242^DL^ os* ; *wupA-2XTY1-sGFP-V5-preTEV-BLRP-3XFLAG* / *wupA-2XTY1-sGFP-V5-preTEV-BLRP-3XFLAG* | <1% |
| ♂ *f^5^ l(1)23437^RL^ os ; wupA-2XTY1-sGFP-V5-preTEV-BLRP-3XFLAG* /+ | √ |
| ♀ *f^5^ l(1)23437^RL^ os / f^5^ l(1)23437^RL^ os ; wupA-2XTY1-sGFP-V5-preTEV-BLRP-3XFLAG* /+ | √ |
| ♀ *f^5^ l(1)23437^RL^ os / f^5^* *l(1)18242^DL^ os ; wupA-2XTY1-sGFP-V5-preTEV-BLRP-3XFLAG* /+ | **†** |
| ♀ *f^5^ l(1)23437^RL^ os / f^5^* *l(1)18242^DL^ os ; wupA-2XTY1-sGFP-V5-preTEV-BLRP-3XFLAG* / *wupA-2XTY1-sGFP-V5-preTEV-BLRP-3XFLAG* | √ |
| ♀ *f^5^ l(1)23437^RL^ os / y w* *l(1)13193^DL^ os ; wupA-2XTY1-sGFP-V5-preTEV-BLRP-3XFLAG* / *wupA-2XTY1-sGFP-V5-preTEV-BLRP-3XFLAG* | √ |
| ♂ *f^5^ hdp^2^ ; wupA-2XTY1-sGFP-V5-preTEV-BLRP-3XFLAG* /+ | Wings up |
| ♂ *y w* *hdp^3^; wupA-2XTY1-sGFP-V5-preTEV-BLRP-3XFLAG* /+ | Wings up |
| **Data of HL regions interaction assay** |  |
| ♀ *y w* *l(1)13193^DL^ os/ Dp(2;1)G146*; +/+ ; +/+ | **†** |
| *♀ f^5^ l(1)18242^DL^ os / Dp(2;1)G146 ; dppH46 wg^Sp^ cn bw /+ ; Ki Sb Dp(1;3)JC153/+* | √ |
| ♀ +/+ ; *dppH46 wg^Sp^ cn bw /+ ; Ki Sb Dp(1;3)JC153/+* | **†** |
| *♀ Dp(2;1)G146 /+ ; +/+ ; Ki Sb Dp(1;3)JC153/+* | √ |
| ♀ *f^5^ l(1)23437^RL^ os / Dp(2;1)G146 ; dppH46 wg^Sp^ cn bw /+* | √ |
| ♂ *y w* *l(1)13193^DL^ os* ; *Dp(1;2) sn^+^72d* /+ ; *Ki Sb Dp(1;3R)JC153/+* | √ |
| *♀ f^5^ l(1)18242^DL^ os / Df(1)hl-a, w cv B; Dp(1;2) sn^+^72d* /+ | **†** |
| ♂ *f^5^* *l(1)18242^DL^ os*; *Dp(1;2) sn^+^72d* /+ | **†** |
| ♀ *y w* *l(1)13193^DL^ os/+ ; Dp(1;2) sn^+^72d* /+ | **†** |
| *♀ f^5^ l(1)18242^DL^ os / Df(1)hl-a, w cv B; Dp(1;2) sn^+^72d* /+ *; Ki Sb Dp(1;3R)JC153/+* | √ |
| ♂ *y w Ubi-GFP l(1)18242^DL^*; *Dp(1;f)LJ9, y^+^* | **†** |
| ♂ *y w* *l(1)13193^DL^ os* ; *Dp(1;f)LJ9, y^+^* | **†** |
| ♂ *y w* *l(1)13193^DL^ os* ; *Dp(1;f)LJ9, y^+^; Ki Sb Dp(1;3R)JC153/+* | √ |

Foot note.- Key to symbols: √ = viable as adult, <1% = Occasional adult escaper, **†** = Lethal, + = normal wing position.
